# Two-Stage SN38 Release from a Core–Shell Nanoparticle Enhances Tumor Deposition and Antitumor Efficacy for Synergistic Combination with Immune Checkpoint Blockade

**DOI:** 10.1021/acsnano.2c09788

**Published:** 2022-11-16

**Authors:** Xiaomin Jiang, Morten Lee, Junjie Xia, Taokun Luo, Jianqiao Liu, Megan Rodriguez, Wenbin Lin

**Affiliations:** † Department of Chemistry, The University of Chicago, 929 East 57th Street, Chicago, Illinois 60637, United States; ‡ Department of Radiation and Cellular Oncology and Ludwig Center for Metastasis Research, The University of Chicago, 5758 South Maryland Avenue, Chicago, Illinois 60637, United States

**Keywords:** prodrug, core−shell nanoparticle, chemoimmunotherapy, tumor microenvironment, antitumor immunity, PD-L1, immunogenic cell death

## Abstract

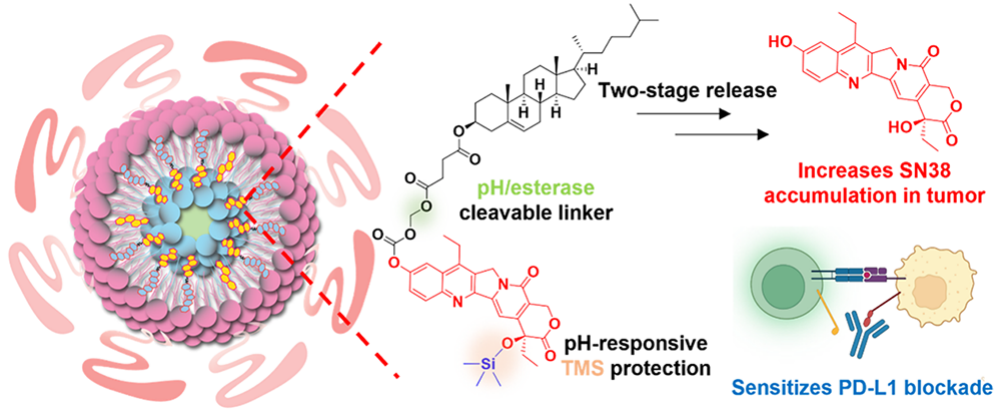

Long-circulating nanomedicines efficiently deliver chemotherapies to tumors to reduce general toxicity. However, extended blood circulation of nanomedicines can increase drug exposure to leukocytes and lead to hematological toxicity. Here, we report a two-stage release strategy to enhance the drug deposition and antitumor efficacy of OxPt/SN38 core–shell nanoparticles with a hydrophilic oxaliplatin (OxPt) prodrug coordination polymer core and a lipid shell containing a hydrophobic cholesterol-conjugated SN38 prodrug (Chol-SN38). By conjugating cholesterol to the phenol group of SN38 via an acetal linkage and protecting the 20-hydroxy position with a trimethylsilyl (TMS) group, Chol-SN38 releases SN38 in two stages via esterase-catalyzed cleavage of the acetal linkage in the liver followed by acid-mediated hydrolysis of the TMS group to preferentially release SN38 in tumors. Compared to irinotecan, OxPt/SN38 reduces SN38 blood exposure by 9.0 times and increases SN38 tumor exposure by 4.7 times. As a result, OxPt/SN38 inhibits tumor growth on subcutaneous, spontaneous, and metastatic tumor models by causing apoptotic and immunogenic cell death. OxPt/SN38 exhibits strong synergy with the immune checkpoint blockade to regress subcutaneous colorectal and pancreatic tumors with 33–50% cure rates and greatly inhibits tumor growth and invasion in a spontaneous prostate cancer model and a liver metastasis model of colorectal cancer without causing side effects. Mechanistic studies revealed important roles of enhanced immunogenic cell death and upregulated PD-L1 expression by OxPt/SN38 in activating the tumor immune microenvironment to elicit potent antitumor immunity. This work highlights the potential of combining innovative prodrug design and nanomedicine formulation to address unmet needs in cancer therapy.

## Introduction

Most chemotherapy regimens administer multiple chemotherapeutics concurrently to cancer patients.^1,2^ This combination therapy strategy promotes synergistic actions of different drugs with distinct anticancer mechanisms to increase therapeutic indices and overcome drug resistance.^3,4^ For example, a combination of folinic acid, fluorouracil, irinotecan (IRI), and oxaliplatin (FOLFIRINOX) increased the median progression-free survival to 6.4 months from 3.3 months for gemcitabine and the median overall survival to 11.1 months from 6.8 months for gemcitabine in patients with advanced pancreatic cancer.^5−8^ These combination regimens often contain both hydrophilic and hydrophobic active drugs, and in some cases, active drugs need to be converted into their prodrugs to enable concurrent administration of the drugs.^9^ For example, in the FOLFIRINOX regimen, the potent topoisomerase I inhibitor SN38 was conjugated to a 1,4′-dipiperidinyl moiety to afford IRI, which increases the aqueous solubility from 11 to 38 μg/mL for SN38 to >20 mg/mL for IRI.^10−12^ However, the esterase-catalyzed conversion of IRI to SN38 occurs mainly in the liver^9^ to release SN38 into the bloodstream,^13^ which causes significant hematological toxicities.

Since the approval of liposomal doxorubicin (Doxil) by the Food and Drug Administration for clinical use in 1995, numerous nanoparticles (NPs) have been developed to deliver chemotherapeutics with improved pharmacokinetic (PK) properties and tumor deposition of drug payloads.^14−18^ The enhanced drug delivery by NPs to tumors has been attributed to passive targeting via the enhanced permeability and retention (EPR) effect, which results from increased neovascularization and poor lymphatic drainage in tumors.^19^ Rationally designed nanomedicines can take advantage of intrinsic differences between normal tissues and tumors,^20−23^ such as acidity, esterase concentrations, and redox potential,^24−28^ to trigger the preferential release of active drugs in tumors. We have developed core–shell nanoscale coordination polymer (NCP) particles for cancer therapy^29−31^ and recently reported OxPt/SN38 NCP particles with a hydrophilic oxaliplatin (OxPt) prodrug coordination polymer core and a lipid shell containing a hydrophobic cholesterol-conjugated SN38 prodrug (Chol-SN38) for effective treatment of colorectal cancer (CRC) in mouse models.^32^ By efficiently delivering OxPt and SN38 to tumors, OxPt/SN38 simultaneously cross-link DNA and inhibit topoisomerase I to significantly inhibit tumor growth and prolong mouse survival without causing serious side effects.

Cancer immunotherapy has enjoyed significant clinical success over the past decade.^33^ In particular, the immune checkpoint blockade (ICB) has afforded durable responses in immunogenic tumors that exhibit high PD-L1 expression and/or have significant preinfiltration of T cells into tumors. For nonimmunogenic tumors, chemotherapies have been used in combination with ICB to elicit antitumor immune responses.^34^ It is believed that chemotherapy can stimulate the tumor immune microenvironment to synergize with ICB. Some chemotherapeutics, including oxaliplatin (OxPt), doxorubicin, and mitoxantrone, have been shown to be proinflammatory by causing immunogenic cell death (ICD), representing ideal candidates for combination therapy with ICB.^35^ Recent studies have also found that topoisomerase I inhibitors such as camptothecin and SN38 can upregulate PD-L1 expression on tumor cells to promote synergy with ICB.^36,37^ We hypothesized that the potent antitumor efficacy of OxPt/SN38 and potential synergy among OxPt, SN38, and ICB could make OxPt/SN38 an ideal nanomedicine combination therapy with ICB to elicit strong antitumor immunity.

Here we elucidate the mechanism for enhanced delivery of SN38 to tumors by OxPt/SN38 and effective chemoimmunotherapy of colorectal, pancreatic, and prostate cancers via the synergistic combination of OxPt/SN38 with an anti-PD-L1 antibody (αPD-L1). Specifically, we discovered that two-stage release of SN38 from the cholesterol-conjugated and trimethylsilyl (TMS)-protected SN38 prodrug (Chol-SN38) significantly enhances drug deposition in tumors. Chol-SN38 first releases SN38-TMS (with the 20-O-TMS moiety) by esterase-catalyzed hydrolysis of the acetal linker in the liver. SN38-TMS is then preferentially hydrolyzed in the acidic tumor microenvironment (TME) to afford a 4.7 ± 1.3 times higher tumor area under curve (AUC) compared to IRI. OxPt/SN38 also increased tumor Pt AUC by 4.0 ± 0.5 fold over OxPt. As a result of enhanced drug depositions in tumors, OxPt/SN38 induced potent ICD of tumor cells and upregulated PD-L1 expression on tumor cells and dendritic cells (DCs) to synergize with αPD-L1 and elicit strong antitumor immunity. OxPt/SN38 plus αPD-L1 cured 33–50% of mice with subcutaneous tumors, inhibited the growth of spontaneous TRAMP prostate tumors by 91.1%, and suppressed liver metastasis of systemic CRC to prolong mouse survival. Mechanistic studies revealed that OxPt/SN38 plus αPD-L1 enhanced tumor immunogenicity, repolarized macrophages to the M1 phenotype, and activated DCs for antigen presentation to cause infiltration of tumor-specific cytotoxic T lymphocytes (CTLs) to tumors. This work highlights the potential of combining rational prodrug design and innovative nanotechnology platforms to develop long-circulating and tumor-responsive nanomedicines for effective chemotherapy and chemoimmunotherapy.

## Results

### Synthesis and Characterization of OxPt/SN38 Particles

**Figure 1:**
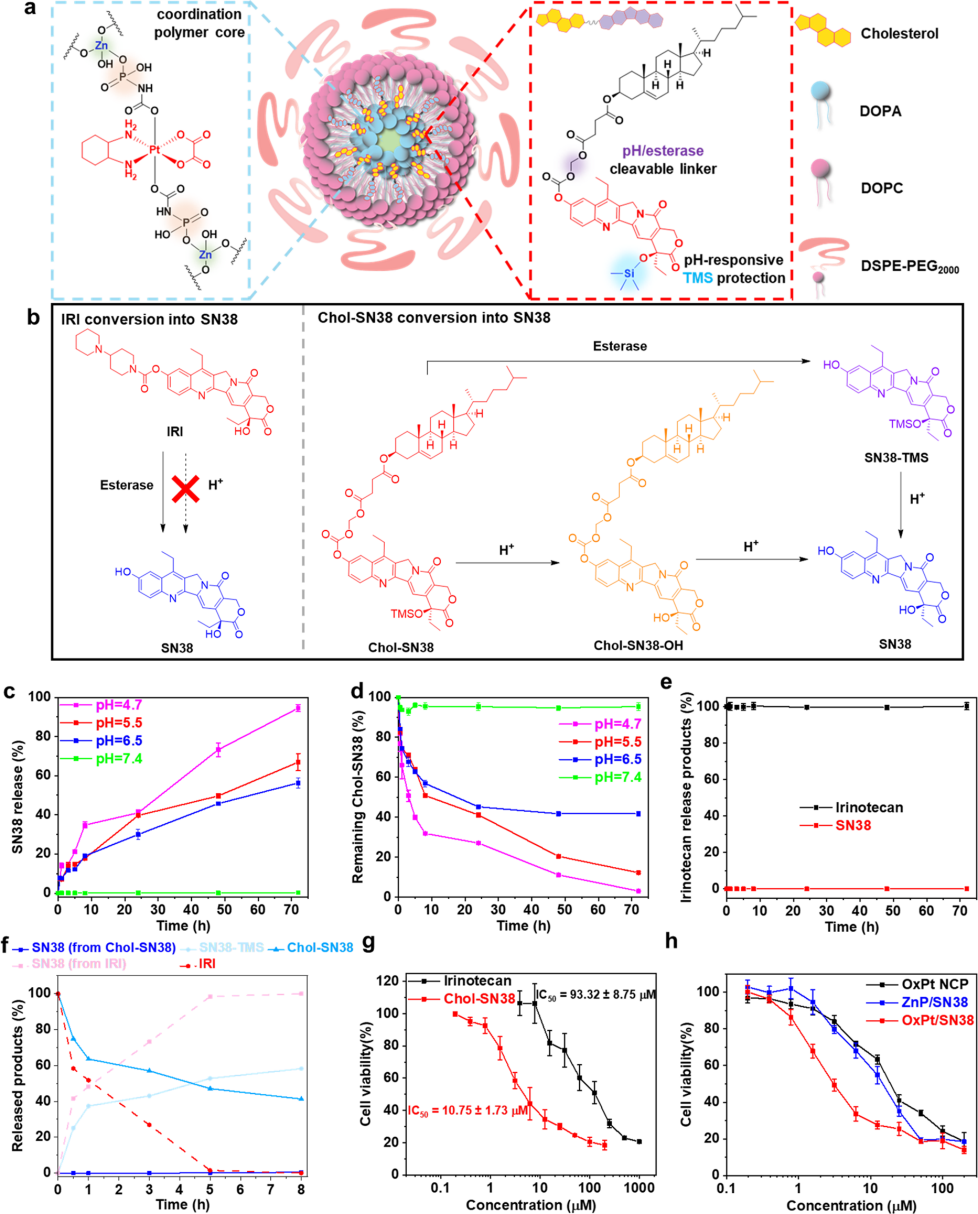
OxPt/SN38 structure, drug release, and cytotoxicity. (a) Schematic illustration of OxPt/SN38 particles with a Zn-(OxPt-bisphosphate) coordination polymer core and a shell of Chol-SN38, cholesterol, DOPC, and DSPE-PEG_2000_. In Chol-SN38, SN38 was doubly modified by conjugation to cholesterol via a cleavable acetal linkage at the phenol position and TMS protection at the 20-hydroxy position. (b) Mechanisms for SN38 conversion from IRI and Chol-SN38. SN38 release (c) and remaining Chol-SN38 (d) from OxPt/SN38 in pH = 4.7, 5.5, 6.5, and 7.4 PBS at 37 °C over 72 h. (e) IRI product conversion in pH = 4.7 PBS at 37 °C over 72 h. (f) Conversion of IRI or Chol-SN38 in OxPt/SN38 to SN38 in esterase-containing PBS (pH = 7.4). (g) MTS assays of IRI and Chol-SN38 on MC38 cells. (h) MTS assays of OxPt NCP, ZnP/SN38, and OxPt/SN38 on MC38 cells.

The OxPt prodrug Pt(dach)(oxalate)(bisphosphoramidic acid) (OxPt-bp) was synthesized as previously described.^30^ The O-20 position of SN38 was first modified with a trimethylsilyl (TMS) group by treating SN38 with *N*,*O*-bis(trimethylsilyl)acetamide to afford SN38-TMS. The O-10 position of SN38-TMS was then conjugated to cholesterol via an acetal linkage to afford Chol-SN38.^32^ The core–shell NCP particle OxPt/SN38 was prepared as reported previously.^32^ In a reverse microemulsion, Zn^2+^ ions first cross-linked OxPt-bp and DOPA to form hydrophobic bare NCP particles (OxPt-bare). Next, OxPt-bare was coated with Chol-SN38 and a lipid mixture of cholesterol, DOPC, and DSPE-PEG_2000_ to produce the core–shell NCP particle OxPt/SN38 (Figure 1a). The OxPt-bare and OxPt/SN38 particles were monodispersed in THF and PBS, respectively, as observed under transmission electron microscopy (TEM, Figure S2a). Dynamic light scattering (DLS) measurements showed *Z*-averaged diameters of 48.3 ± 1.2 and 109.9 ± 5.2 nm and polydispersity indexes (PDIs) of 0.16 ± 0.02 and 0.17 ± 0.03 for OxPt-bare and OxPt/SN38, respectively (Figure S2b).

### Tumor-Specific Drug Release from OxPt/SN38

We elucidated the mechanism for the enhanced SN38 tumor deposition by OxPt/SN38 over IRI. We first compared the release profiles of SN38 from IRI and OxPt/SN38 under different conditions to simulate the TME (low pH) and the liver environment (high esterase level) (Figure 1b). We evaluated drug release from Chol-SN38 in OxPt/SN38 at pH = 4.7 (mimicking lysosomal conditions) and at pH = 5.5 and 6.5 (mimicking TME and endosomal conditions, respectively). At pH = 4.7 PBS at 37 °C, both TMS and the acetal linkage on Chol-SN38 were cleaved to release 95% of SN38 in 72 h, while IRI was completely stable without producing SN38 in 72 h (Figure 1c–e). Totals of 67% and 56% SN38 were released from OxPt/SN38 at pH = 5.5 and 6.5, respectively, over 72 h. In comparison, at pH = 7.4, only 2% SN38 was released from OxPt/SN38. This result is supported by the quantification of Chol-SN38 under these conditions. Totals of 3%, 12%, 42%, and 95% Chol-SN38 remained at pH = 4.7, 5.5, 6.5, and 7.4, respectively, at 72 h (Figure 1d). We further quantified the amount of Chol-SN38-OH that was produced via hydrolysis of the −OTMS group under different conditions (Figure S2c). At low pH, Chol-SN38-OH first formed and then was further hydrolyzed to release SN38. These results demonstrated that Chol-SN38 could preferentially release SN38 in acidic TME, but IRI could not be triggered to release SN38 at low pH.

In PBS with 10 unit/mL esterase (pH = 7.4, 37 °C), OxPt/SN38 gradually released SN38-TMS over time with 58% SN38-TMS and 41% Chol-SN38 being detected in 8 h (Figure 1f). Approximately 1% SN38 was released under this condition in 8 h. In contrast, 48% and 100% IRI were converted to SN38 under this condition in 1 and 8 h, respectively. This result suggests that OxPt/SN38 releases SN38-TMS only in the liver and minimizes SN38 exposure in blood circulation. As SN38-TMS is at least 10 times less toxic than SN38 (Table S1), OxPt/SN38 has the potential to minimize hematological toxicity, which is a dose-limiting toxicity for IRI-based regimens. To confirm hydrolysis of the 20-O-TMS group, we incubated SN38-TMS under different conditions. SN38-TMS slowly released SN38 in pH = 7.4 PBS but quickly released SN38 in pH = 4.7 PBS, resulting in the release of 75% SN38 at pH = 4.7 in 24 h (Figure S2d). This result suggests that, in the esterase-abundant environment in tumors,^38^ Chol-SN38 delivered by OxPt/SN38 first releases SN38-TMS by esterase and low pH. SN38-TMS is further hydrolyzed to SN38 at low pH. Compared to esterase-triggered release of SN38 from IRI, the dual activation mechanism of SN38 from OxPt/SN38 in tumors further minimizes SN38 exposure to normal tissue.

### Synergistic Cytotoxicity of OxPt/SN38

We next examined the *in vitro* cytotoxicity of IRI, Chol-SN38, and OxPt/SN38 on MC38 murine colon carcinoma cells by an MTS assay. After 48 h of incubation, Chol-SN38 exhibited 9-fold higher cytotoxicity than IRI against MC38 cells. The IC_50_ values were 10.75 ± 1.73 and 93.32 ± 8.75 μM for Chol-SN38 and IRI, respectively (Figure 1g). We also probed the synergy between OxPt and SN38 in NCP particles by an MTS assay. The combination of OxPt and SN38 prodrugs in OxPt/SN38 significantly reduced the OxPt IC_50_ value from 14.24 ± 1.32 μM for OxPt NCP to 2.92 ± 0.44 μM for OxPt/SN38 and the Chol-SN38 IC_50_ value from 10.86 ± 1.24 μM for ZnP/SN38 to 5.26 ± 0.79 μM for OxPt/SN38 on MC38 cells (Figure 1h and Table S1). These results support the superiority of Chol-SN38 over IRI as a prodrug for SN38 and a strong synergy between OxPt and SN38 delivered by OxPt/SN38.

### TMS Modification of SN38 Prodrug Minimizes SN38 Blood Exposure

**Figure 2:**
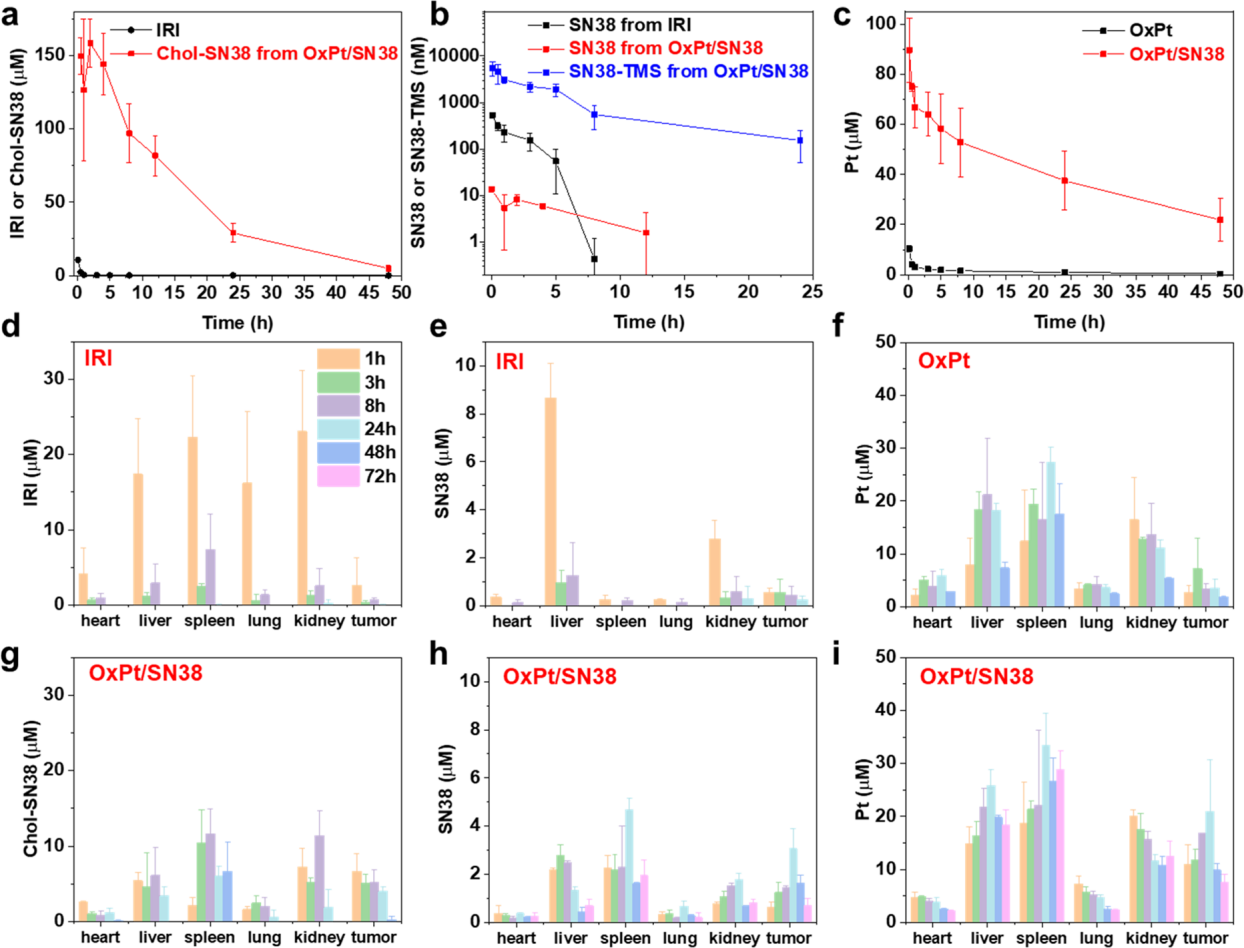
Pharmacokinetics and biodistribution of OxPt/SN38. PK profiles of IRI or Chol-SN38 from OxPt/SN38 (a), SN38 from IRI or SN38-TMS and SN38 from OxPt/SN38 (b), and Pt (c) in rat plasma after an i.v. injection of OxPt plus IRI or OxPt/SN38 at doses of 2.0 mg/kg OxPt and 3.6 mg/kg SN38 equiv. Time-dependent accumulation of SN38 prodrugs (d, g), SN38 (e, h), and Pt (f, i) after an i.v. injection of OxPt plus IRI or OxPt/SN38 at 3.5 mg/kg OxPt and 6.2 mg/kg SN38 equiv) to MC38 tumor-bearing mice.

To investigate the impact of TMS modification of SN38 prodrug on blood circulation behaviors of OxPt/SN38, we performed PK studies of IRI plus OxPt and OxPt/SN38 on Sprague–Dawley rats. With stable pegylation, OxPt/SN38 effectively resisted uptake by the monophagocytic system and prevented renal clearance to significantly increase blood exposure (areas under curves, AUCs) of OxPt and SN38 prodrugs by 21.0 and 129.3 times, respectively, over OxPt plus IRI, after intravenous (i.v.) injections to healthy Sprague–Dawley rats (Figure 2a,c, Table 1, and Tables S2–S4). OxPt/SN38 also increased the Pt half-life from 0.90 ± 0.32 h for OxPt to 30.84 ± 4.95 h for OxPt/SN38 and the SN38 prodrug half-life from 2.71 ± 1.82 h for IRI to 9.74 ± 1.00 h for OxPt/SN38. Importantly, SN38-TMS was found to be a major circulating metabolite in the blood for OxPt/SN38 (Figure 2b, Table 1, and Table S3). Compared to IRI, OxPt/SN38 reduced SN38 AUC_0-inf_ by 9 times (from 1.30 ± 0.53 μM h for IRI to 0.15 ± 0.04 μM h for OxPt/SN38) and reduced SN38 *C*_max_ by 40 times (from 0.61 ± 0.02 μM for IRI to 0.02 ± 0.01 μM for OxPt/SN38). These results indicate that TMS modification of the SN38 prodrug minimizes SN38 blood exposure by changing the circulating active metabolite from SN38 to SN38-TMS.

**Table 1:** Pharmacokinetics of OxPt plus IRI and OxPt/SN38 on Sprague–Dawley Rats^a^

drug	analyte	*t*_1/2_ (h)	C_max_ (μM)	AUC_0-inf_ (μM h)
OxPt	Pt	0.90 ± 0.32	25.80 ± 2.98	140.54 ± 42.34
				
irinotecan	irinotecan	2.71 ± 1.82	1.34 ± 0.37	9.29 ± 3.60
	SN38	1.87 ± 1.34	0.61 ± 0.02	1.30 ± 0.53
				
OxPt/SN38	Pt	30.84 ± 4.95	93.13 ± 16.15	2946.28 ± 1062.03
	Chol-SN38	9.74 ± 0.99	170.53 ± 14.10	1211.76 ± 26.19
	SN38-TMS	4.03 ± 1.19	6.59 ± 2.14	25.99 ± 4.92
	SN38	4.22 ± 1.28	0.02 ± 0.01	0.15 ± 0.04

a PK was performed by a single intravenous injection of OxPt plus irinotecan or OxPt/SN38 at 2.0 mg/kg OxPt and 3.6 mg/kg SN38 equiv with noncompartmental analysis.

### TMS Modification of SN38 Prodrug Enhances SN38 Delivery to Tumors

We also conducted biodistribution studies of OxPt plus IRI and OxPt/SN38 in MC38 tumor-bearing mice to investigate the delivery efficiency of OxPt/SN38. Due to its long circulation nature, OxPt/SN38 showed a higher Chol-SN38 tumor AUC than IRI, while lowering its concentration in normal organs (Figure 2d,g). As IRI is water-soluble and easily cleaved by liver esterase, i.v. injection of IRI led to high concentrations of SN38 in livers and kidneys (Figure 2e). In contrast, OxPt/SN38 did not show high Chol-SN38 or SN38 exposure in livers or kidneys but instead accumulated in tumors with a 4.9 ± 1.3 fold higher SN38 AUC than IRI (Figure 2h and Table S5). This is likely due to the circulation of SN38-TMS in the blood, which preferentially hydrolyzes to SN38 in the acidic TME. OxPt/SN38 also showed a 4.0 ± 0.5 times higher tumor Pt AUC than OxPt without increasing Pt concentrations in other organs (Figure 2f,i). The Pt concentrations in tumors remained above the IC_50_ values of CT26 and MC38 cells for a period of 72 h (Table S1). In comparison, OxPt gave a tumor Pt concentration at or above the IC_50_ value only at 3 h post i.v. injection. These results show that OxPt/SN38 markedly increases the concentrations of OxPt and SN38 in tumors due to its long circulation in the blood and the preferential hydrolysis of SN38-TMS to SN38 in the TME.

### OxPt/SN38 Upregulates PD-L1 and Induces Immunogenic Cell Death

**Figure 3:**
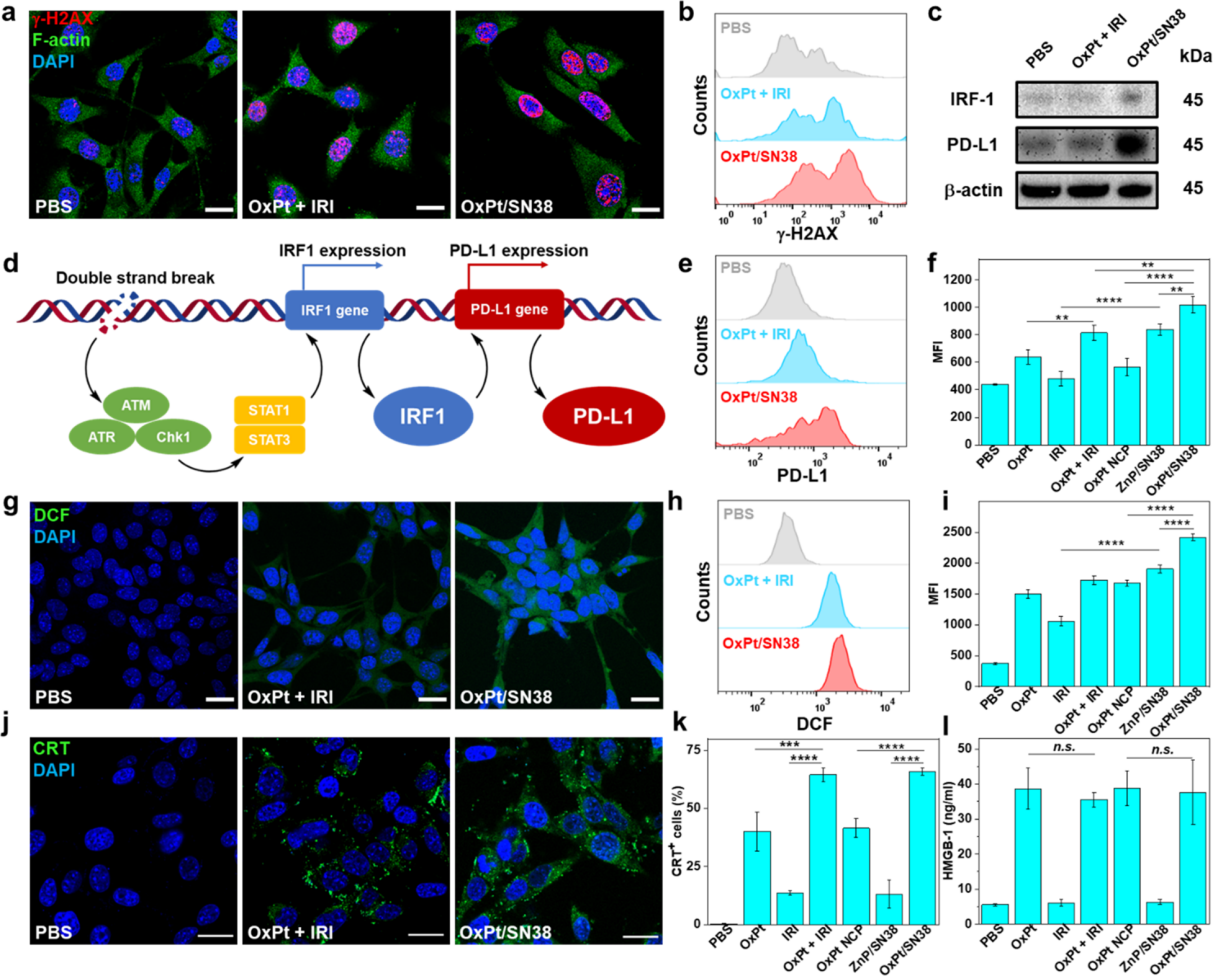
OxPt/SN38 induces ICD and upregulates PD-L1. CLSM images (a) and flow cytometry (b) showing DNA DSBs of MC38 cells after treatment with OxPt plus IRI or OxPt/SN38. Scale bar: 20 μm. Cellular morphology was visualized by F-actin (green) staining. (c) Western blot of γ-H2AX, IRF1, and PD-L1 of MC38 cells. (d) Schematic showing PD-L1 upregulation induced by DNA DSBs. DSBs activate STAT signaling through ATM/ATR/Chk1 kinases, which in turn induces IRF1 activation and downstream PD-L1 upregulation. Flow cytometry (e) with statistical analysis (f) of PD-L1 expression in MC38 cells, *n* = 3. CLSM images (g) and flow cytometry (h) with statistical analysis (i) of ROS generation in MC38 cells, *n* = 3. CLSM images (j) and flow cytometry with statistical analysis (k) of CRT expression in MC38 cells, *n* = 3. (l) HMGB-1 release from MC38 cells detected by ELISA, *n* = 3. In all treatments, MC38 cells were incubated with drugs at 5 μM OxPt equiv and 9 μM SN38 equiv for 24 h. Data are expressed as means ± SD, **p < 0.01, ***p < 0.001, and ****p < 0.0001, by one-way ANOVA with Tukey’s multiple comparisons test. MFI denotes mean fluorescent intensity.

Many chemotherapies are known to upregulate PD-L1 in tumors by causing DNA double-strand breaks (DSBs).^39,40^ It is known that DSBs can activate STAT signaling through ataxia telangiectasia mutated (ATM)/ataxia telangiectasia and Rad3-related protein (ATR)/checkpoint kinase 1 (Chk1) kinases.^39^ The activation of STAT1 and STAT3 induces interferon regulatory factor 1 (IRF1) and its downstream PD-L1 upregulation (Figure 3d).^39^ We first found that OxPt/SN38 induced strong DSBs in MC38 cells. CLSM and flow cytometry results showed that OxPt/SN38 induced 26% higher γ-H2AX signals than OxPt plus IRI. All combination groups exhibited higher γ-H2AX signals than monotherapy treatments (Figure 3a,b and Figures S3 and S4). Western blot showed that OxPt/SN38 upregulated both IRF-1 and PD-L1 by 2-fold over OxPt plus IRI at 24 h post treatments (Figure 3c). Flow cytometry results showed that OxPt/SN38 induced 25% higher PD-L1 signals than OxPt plus IRI and combination groups induced higher PD-L1 signals than monotherapy treatments (Figure 3e,f and Figure S5). These results show that OxPt/SN38 efficiently and synergistically induce DSBs to upregulate PD-L1 via the DSB-IRF1-PD-L1 axis.

As cancer cells are sensitive to oxidative stress, we investigated the ability of OxPt/SN38 to generate reactive oxygen species (ROS). ROS can cause apoptotic cell death by directly reacting with membranes, DNAs, proteins, and organelles or generating secondary products to damage biomolecules and organelles. Both OxPt and SN38 induced ROS generation in tumor cells, but the ROS signal significantly increased when OxPt and SN38 were given in combination (Figure 3g-I and Figures S6 and S7). Chol-SN38 was also more efficient in generating ROS than IRI. OxPt/SN38 showed a 40% higher ROS signal than OxPt plus IRI.

ER stress and ROS production are key intracellular pathways for ICD induction, which activates danger signaling pathways by trafficking damage-associated molecular patterns (DAMPs) to the extracellular space.^41−44^ We showed that both OxPt and OxPt/SN38 induced ICD with cell-surface exposure of calreticulin (CRT) (Figure 3j,k and Figure S8a). We also performed CRT fluorescence staining of cryo-sectioned slides of tumors. While OxPt or OxPt plus IRI did not increase CRT signals *in vivo* (Figure S8b), OxPt/SN38 elicited significant CRT exposure in the tumors. We further determined the release of high mobility group box-1 (HMGB-1) protein from cells treated with OxPt and OxPt/SN38 by an enzyme-linked immunosorbent assay (ELISA). Incubation with OxPt/SN38 caused significant release of HMGB-1 from MC38 cells (Figure 3l).

### OxPt/SN38 Elicits Antitumor Effects and Synergizes with ICB

The antitumor efficacy of OxPt/SN38 was first studied in subcutaneous MC38 and CT26 murine CRC tumor models and a subcutaneous KPC murine pancreatic tumor model. Mice were i.v. injected with OxPt/SN38, OxPt plus IRI, OxPt NCP, ZnP/SN38, or PBS at 3.5 mg/kg OxPt and 6.2 mg/kg SN38 equiv or/and i.p. injected with 75 μg of αPD-L1 once every 3 days (Q3D).

**Figure 4:**
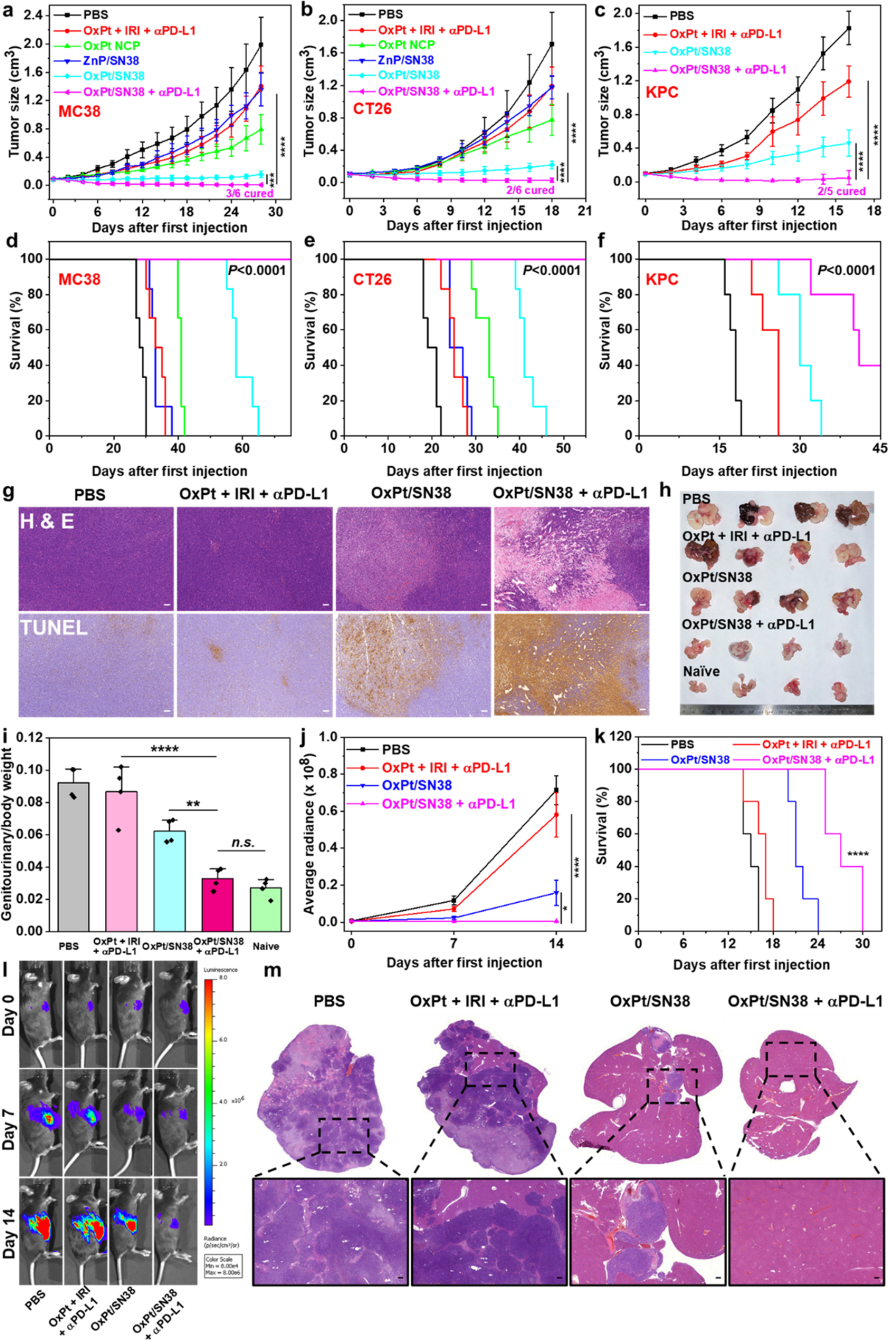
*In vivo* anticancer efficacy. Tumor growth curves (a) and survival curves (d) of MC38 tumor-bearing C57BL/6 mice after indicated treatments, *n* = 6. Tumor growth curves (b) and survival curves (e) of CT26 tumor-bearing BALB/c mice after indicated treatments, *n* = 6. Tumor growth curves (c) and survival curves (f) of KPC tumor-bearing C57BL/6 mice after indicated treatments, *n* = 5. PBS, OxPt plus IRI, OxPt NCP, ZnP/SN38, or OxPt/SN38 was intravenously dosed once every 3 days with 3.5 mg/kg OxPt or and 6.2 mg/kg SN38 equiv to the MC38 model (eight doses) and CT26 and KPC models (six doses). A 75 μg portion of anti-PD-L1 antibody (αPD-L1) was i.p. injected in the same dosing schedule. (g) H&E staining and TUNEL IHC staining of excised MC38 tumors at the end point. Scale bars: 100 μm. Photo (h) and ratios of genitourinary tract weights versus total body weights (i) of TRAMP mice treated with PBS, OxPt plus IRI plus αPD-L1, OxPt/SN38, or OxPt/SN38 plus αPD-L1 at an equivalent dose of 3.5 mg/kg OxPt and/or 6.2 mg/kg SN38 Q3D for ten doses, *n* = 4. MC38-OVA-luciferase luminescence intensities (j), survival curves (k), and *in vivo* imaging (l) of C57BL/6 mice with liver metastases at different time points. Seven days after injection of MC38-OVA-luciferase cells into the spleens, mice were treated with PBS, OxPt plus IRI plus αPD-L1, OxPt/SN38, or OxPt/SN38 plus αPD-L1 at an equivalent dose of 3.5 mg/kg OxPt and/or 6.2 mg/kg SN38 Q3D for 5 doses, *n* = 5. (m) Representative images of H&E staining of livers of C57BL/6 mice with liver metastases at the end point. Scale bars: 100 μm. Data are expressed as means ± SD. The data were analyzed by Student’s two-tailed *t* test (a, b) or one-way analysis of variance (ANOVA; c, i–k). *p < 0.05, **p < 0.01, ***p < 0.001, ****p < 0.0001. A log-rank (Mantel–Cox) test was used for statistical analysis of survival curves.

After eight injections of OxPt/SN38 plus αPD-L1, MC38 tumor-bearing C57BL/6 mice showed 99.6% tumor growth inhibition (TGI) with a 50% cure rate. The antitumor efficacy of OxPt/SN38 plus αPD-L1 was superior to the combination treatment of OxPt, IRI, and αPD-L1, which provided a TGI of 29.3% (Figure 4a). The synergy between OxPt/SN38 and αPD-L1 allowed eradication of tumors in 50% of the mice (Figure 4d). Although OxPt/SN38 alone showed strong antitumor efficacy with a TGI of 92.1%, no mice were cured in this group. Furthermore, H&E and TUNEL staining revealed more severe tumor apoptosis/necrosis for OxPt/SN38 plus αPD-L1 treatment (Figure 4g). OxPt NCP and ZnP/SN38 monotherapy nanoparticles showed slight tumor growth inhibition with TGI values of 60.2% and 31.8%, respectively.

For the CT26 model, OxPt/SN38 plus αPD-L1 also showed potent anticancer activity with a TGI of 98.6% and a 33.3% cure rate. In comparison, the combination of OxPt, irinotecan, and αPD-L1 provided a modest TGI of 30.4% (Figure 4b,e). With a TGI of 87.3%, OxPt/SN38 showed a strong synergy between the two drugs released from the nanoparticle to significantly enhance the anticancer efficacy over OxPt NCP with a TGI of 54.7% and ZnP/SN38 with a TGI of 31.3%. OxPt/SN38 sensitized the tumors to αPD-L1 treatment, which could not be achieved with the free drug combination.

We also evaluated the antitumor efficacy on a murine pancreatic KPC tumor model. KPC cells develop “cold” tumors with a high percentage of immunosuppressive cells.^45^ OxPt/SN38 plus αPD-L1 showed 97.4% tumor growth inhibition with a 40.0% cure rate, while the combination of OxPt, IRI, and αPD-L1 exhibited a modest TGI of 34.8% (Figure 4c). OxPt/SN38 only showed a moderate TGI of 74.9%. These results indicate that OxPt/SN38 efficiently inhibits tumor growth and synergizes with ICB to eradicate tumors in some mice (Figure 4f). OxPt/SN38 treatment was well tolerated by both BALB/c and C57BL/6 mice, as judged by body weight change patterns, hematology analysis, and histology of major organs including heart, liver, spleens, lungs, and kidneys (Figures S9 and 10).

We further evaluated the antitumor efficacy of OxPt/SN38 plus αPD-L1 on the C57BL/6-Tg(TRAMP)8247Ng/J (TRAMP) spontaneous prostate cancer model.^46^ TRAMP mice develop spontaneous autochthonous prostate tumors which closely resemble human prostate cancer pathogenesis starting at ∼10 weeks of age. They start to develop metastases in the lymph nodes, lungs, and occasionally other organs at ∼12 weeks.^47^ TRAMP mice were verified by genotyping, and 24-week-old TRAMP mice were used for antitumor efficacy studies. After the mice were treated with 10 doses of PBS, OxPt/SN38, OxPt plus IRI plus αPD-L1, or OxPt/SN38 plus αPD-L1, the prostate and seminal vesicles were dissected and photographed 30 days after the cessation of treatments (Figure 4h,i). Genitourinary tract/body weight ratios showed that OxPt/SN38 plus αPD-L1 prevented tumor formation in genitourinary tracts, reduced tumor burden by 91.1% compared to PBS control, and showed very little difference from naïve wild type mice. However, the combination of OxPt, IRI, and αPD-L1 only reduced the tumor burden by 8.6%, while OxPt/SN38 reduced the tumor burden by 45.8%, in comparison to PBS control. This result supports the strong synergy between OxPt/SN38 and αPD-L1 in inhibiting the growth of spontaneously developed prostate tumors that closely mimic human prostate cancer.

We established a metastatic model of CRC by intrasplenic injection of MC38-luciferase cells to investigate the antimetastatic activity of OxPt/SN38 plus αPD-L1. Beginning 7 days after intrasplenic injection of MC38-luciferase cells, the mice were treated with OxPt/SN38 plus αPD-L1, OxPt plus IRI plus αPD-L1, OxPt/SN38, or PBS once every 3 days and injected weekly with luciferin, anesthetized with 2% isoflurane (v/v), and imaged on an IVIS system to visualize tumors and quantify tumor burdens (Figure 4j–l). While the free drug combination had little effect on the tumor burden compared to the PBS group, OxPt/SN38 treatment significantly inhibited tumor growth, as determined by the bioluminescence signals. Treatment with OxPt/SN38 plus αPD-L1 reduced the bioluminescence signal from the baseline at day 14 after the first dose. Fourteen days post treatment, the mice were euthanized to assess liver metastasis by H&E staining, which showed a significant decrease of tumor cell invasion in the OxPt/SN38 plus αPD-L1 group (Figure 4m). Treatment with OxPt/SN38 plus αPD-L1 also prolonged the median survival from 15 days to 27 days after the first treatment. In comparison, treatments with OxPt/SN38 and OxPt plus IRI plus αPD-L1 increased the median survival by 6 and 2 days, respectively. These results demonstrate the synergistic effects of OxPt/SN38 and αPD-L1 on inhibition of liver metastases of CRC.

We tested the tolerability of OxPt/SN38 on female Sprague–Dawley rats at 4.0 mg/kg OxPt and 18.3 mg/kg Chol-SN38 on a once every week schedule for four doses. This dose is equivalent to Q3D administration of OxPt/SN38 to mice at 3.5 mg/kg OxPt for ten doses based on body surface area scaling. OxPt/SN38 did not impact the body weight change pattern of the rats in comparison to PBS control (Figure S11). The rats treated with four doses of OxPt/SN38 did not show any decrease in neutrophil counts or increases in liver enzymes, creatinine, and blood urea nitrogen over the rats treated with PBS (Table S6). These results show that the therapeutic dose of OxPt/SN38 is well tolerated in rats without causing hematological, liver, and kidney toxicities.

### OxPt/SN38 Enhances Antitumor Immunity by Sensitizing Tumor Cells to ICB

**Figure 5:**
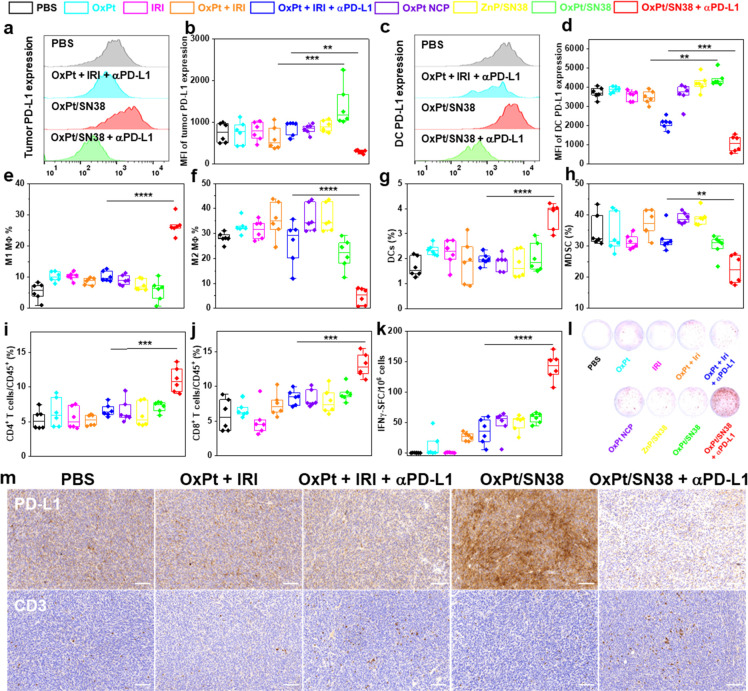
OxPt/SN38 and αPD-L1 synergistically remodel the TME to elicit antitumor immunity. Flow cytometry with statistical analysis of PD-L1 expression on MC38 tumor cells (a, b) and DCs (c, d) 4 days after treatment of MC38 tumor-bearing C57BL/6 mice with 2 doses of PBS, OxPt, IRI, OxPt plus IRI, OxPt plus IRI plus αPD-L1, OxPt NCP, ZnP/SN38, OxPt/SN38, OxPt/SN38 plus αPD-L1, *n* = 6. (e–h) Immune cell infiltration into the tumors from mice in (a)–(d) determined by fluorescence-activated cell sorting (FACS). The subpopulations were defined: M1 macrophages as CD45^+^CD11b^+^F4/80^+^CD86^+^ (e); M2 macrophages as CD45^+^CD11b^+^F4/80^+^CD206^+^ (f); DCs as CD45^+^CD11b^+^CD11c^+^ (g); MDSCs as CD45^+^CD11b^+^GR-1^+^F4/80^–^ (h), *n* = 6. T cell infiltration into the tumors (i, j) and ELISpot assay (k, l) of MC38 tumor bearing C57BL/6 mice on day 13 after five doses of OxPt/SN38 plus αPD-L1, OxPt plus IRI plus αPD-L1, OxPt/SN38, or PBS. Helper T cells were defined as CD45^+^CD3e^+^CD4^+^ (i). Cytotoxic T cells were defined as CD45^+^CD3e^+^CD8^+^ (j), *n* = 6. (m) PD-L1 and CD3 IHC staining of excised MC38 tumors after eight doses of PBS, OxPt plus IRI, OxPt plus IRI plus αPD-L1, OxPt/SN38, or OxPt/SN38 plus αPD-L1. Scale bars: 100 μm. Data are expressed as means ± SD, **p < 0.01, ***p < 0.001, ****p < 0.0001, by one-way ANOVA with Tukey’s multiple comparisons test.

We determined immune cell infiltration into tumors to probe the changes in the TME. When MC38 tumors reached 80–120 mm^3^ in size, mice were treated with PBS, OxPt, IRI, OxPt plus IRI, OxPt plus IRI plus αPD-L1, OxPt NCP, ZnP/SN38, OxPt/SN38, or OxPt/SN38 plus αPD-L1 and analyzed for innate immunity 4 days after the first treatment and for adaptive immunity 13 days after the first treatment on a Q3D schedule. We first evaluated PD-L1 expression levels on tumor cells (CD45^–^) and DCs (CD45^+^CD11b^+^CD11c^+^). Consistent with the *in vitro* results, OxPt/SN38 upregulated PD-L1 expression on MC38 tumor cells with 1.8 times higher PD-L1 expression than the PBS group (Figure 5a,b). Different from *in vitro* results, free OxPt, IRI, or OxPt plus IRI did not show any effect on tumor PD-L1 expression due to their poor pharmacokinetic behaviors. While no obvious change of PD-L1 expression was observed for OxPt plus IRI plus αPD-L1, the addition of αPD-L1 to OxPt/SN38 significantly reduced the PD-L1 expression level on tumor cells from both OxPt/SN38 and PBS groups. The OxPt/SN38 plus αPD-L1 group exhibited 60% lower PD-L1 expression on tumor cells than the PBS group. A similar trend was also found for DCs, another important subset of immune cells with PD-L1 expression (Figure 5c,d). OxPt/SN38 upregulated PD-L1 expression on DCs by 20%, while free OxPt, IRI, and OxPt plus IRI did not show a significant difference from PBS. The addition of αPD-L1 to OxPt/SN38 treatment significantly reduced PD-L1 expression on DCs with 72% lower PD-L1 expression than the PBS group. Consistent with the flow cytometry results, tumor immunohistochemistry (IHC) staining of the PD-L1 marker revealed that the OxPt/SN38 group had a much higher PD-L1 expression level than the PBS group while the PD-L1 expression level in the OxPt/SN38 plus αPD-L1 group was lower than those in both PBS and free drug combination groups (Figure 5m).

OxPt/SN38 plus αPD-L1 treatment drastically increased surface expression of CD86 on macrophages (MΦ) (26.4 ± 2.9% vs 5.3 ± 2.8% for PBS) while decreasing surface expression of CD206 on macrophages (4.6 ± 2.8% vs 28.5 ± 2.3% for PBS) (Figure 5e,f). As CD86 and CD206 are markers for M1 and M2 macrophages, respectively, these results suggest repolarization of M2 macrophages to M1 macrophages and/or recruitment of inflammatory M1 macrophages by OxPt/SN38 plus αPD-L1 treatment. The OxPt/SN38 plus αPD-L1 group also showed a marked increase of DCs in tumors (3.6 ± 0.6% vs 1.8 ± 0.4% for PBS) (Figure 5g). In F4/80-myeloid cells, the percentage of immunosuppressive myeloid-derived suppressor cells (MDSCs) decreased from 34.8 ± 6.1% for the PBS group to 23.7 ± 5.2% for the OxPt/SN38 plus αPD-L1 group (Figure 5h). In contrast, there is an obvious difference between the PBS control and OxPt, IRI, OxPt plus IRI, OxPt plus IRI plus αPD-L1, and monotherapy nanoparticles.

The percentages of helper (CD4^+^) and cytotoxic (CD8^+^) T cells in leukocytes (CD45^+^) increased in the tumors treated with OxPt/SN38 plus αPD-L1 (Figure 5i,j). OxPt/SN38 plus αPD-L1 and PBS groups showed CD4^+^ T cell populations of 11.1 ± 1.8% and 5.3 ± 1.4%, respectively, and CD8 + T cell populations of 13.1 ± 1.7% and 5.9 ± 2.3%, respectively. Tumor IHC staining of the CD3 marker revealed significantly increased infiltration of T cells in the OxPt/SN38 plus αPD-L1 group over other treatment groups (Figure 5m). We also detected tumor antigen-specific CD8^+^ T cells in leukocyte-abundant spleens by an enzyme-linked immunospot (ELISpot) assay (Figure 5k,l). Splenocytes were obtained from MC38 tumor-bearing C57BL/6 mice and stimulated with CD8^+^ T cell specific epitope KSPWFTTL for 48 h. The mice treated with OxPt/SN38 plus αPD-L1 showed significantly more antigen-specific IFN-γ-producing T cells. In contrast, OxPt plus IRI plus αPD-L1 only slightly increased IFN-γ-producing T cells over PBS. These results demonstrate that, in combination with αPD-L1, OxPt/SN38 elicits superior antitumor immunity over the free drug combination by engaging both innate and adaptive immune responses.

## Discussion

Hydrophobic organic drugs are typically converted into prodrugs with increased aqueous solubility.^9^ Nearly half of all marketed drugs are prodrugs containing hydrolyzable ester and carbamate linkages that are activated by esterases in the liver.^48^ For example, IRI contains a hydrolyzable carbamate linkage to the 1,4′-dipiperidinyl moiety to increase the aqueous solubility from 11 to 38 μg/mL for SN38 to >20 mg/mL for IRI. The conversion of IRI to SN38 in the liver exposes leukocytes to high SN38 concentrations in the blood, leading to severe hematological toxicity in many patients receiving IRI-based chemotherapy regimens.^9^ Nanotechnology provides an opportunity to design prodrugs that can be preferentially released in tumors.^49−51^ We discovered two-stage release of SN38 from Chol-SN38 delivered by OxPt/SN38 to significantly enhance drug deposition in tumors. Chol-SN38 first releases SN38-TMS via esterase-catalyzed hydrolysis of the acetal linker in the liver. SN38-TMS is then preferentially hydrolyzed in the acidic TME to afford a 4.9 ± 1.3-times higher tumor area under curve (AUC) compared to IRI. The enhanced delivery of both SN38 and OxPt to tumors by OxPt/SN38 elicited potent antitumor efficacy in multiple tumor models without causing side effects.

The PD-1/PD-L1 blockade can restore T cell cytotoxicity against tumor cells. High PD-L1 expression and/or pre-existing tumor-infiltrating T cells are essential for ICB to be effective. Increasing evidence has showed that chemotherapies such as OxPt and SN38 derivatives (including IRI) can activate TMEs to synergize with ICB.^36,37^ Our study shows that OxPt/SN38 elicits strong synergy with αPD-L1 to regress subcutaneous colorectal and pancreatic tumors with 33–50% cure rates. OxPt/SN38 also inhibits tumor growth in a spontaneous prostate cancer model and prevents tumor invasion in a liver metastasis model of colorectal cancer.

**Figure 6:**
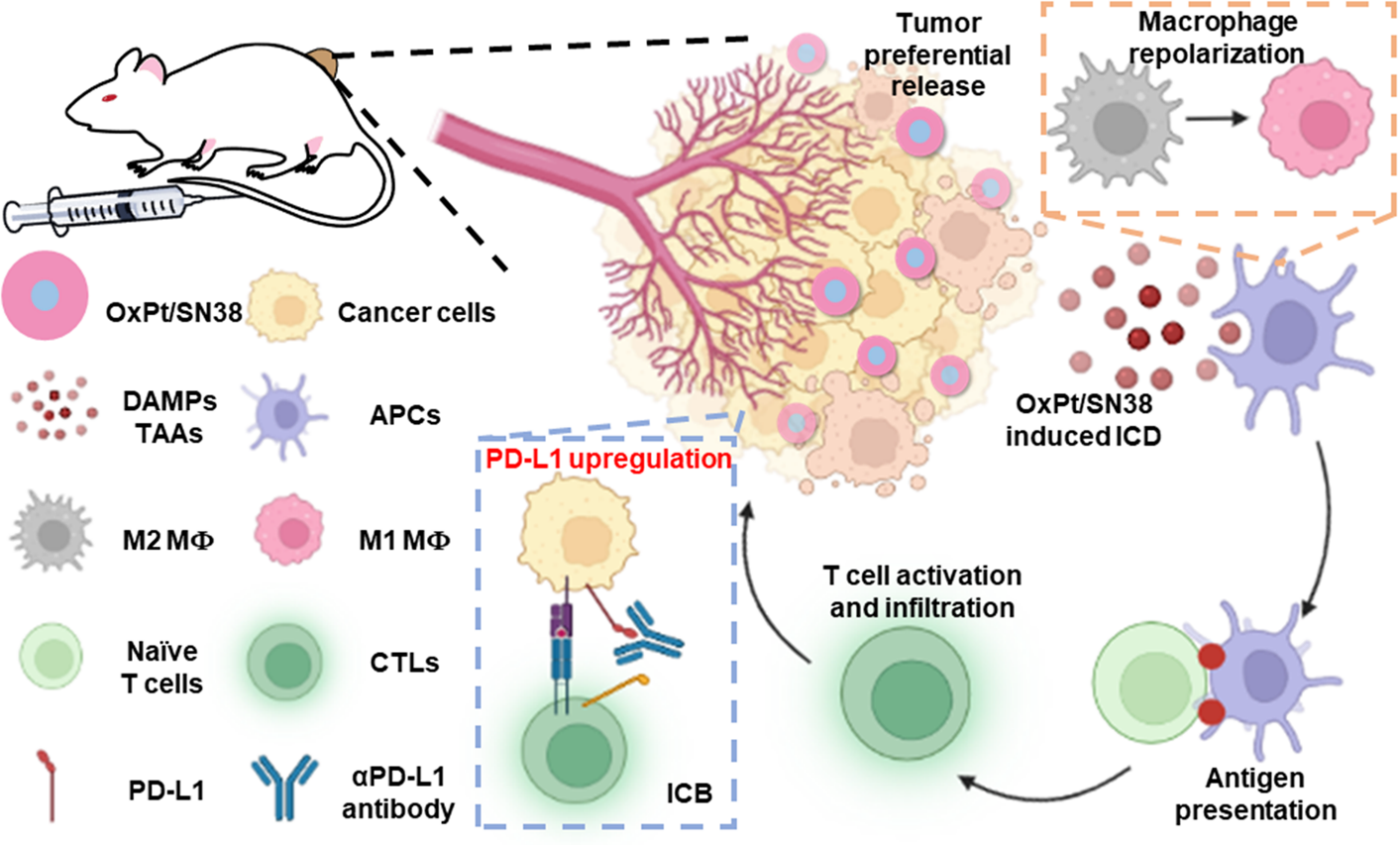
Proposed anticancer mechanisms and immune activation pathways. Intravenously injected OxPt/SN38 preferentially releases active drugs in the acidic TME. OxPt/SN38 not only kills cancer cells by inhibiting DNA replication but also induces severe ICD and upregulates PD-L1 expression on tumor cells and DCs. CRT expression on tumor cell surface and release of DAMPs, such as HMGB1, facilitate phagocytosis of dying tumor cells by APCs for antigen processing and presentation. Upon migration to lymph nodes, DCs activate naïve T cells. The repolarization of macrophages from M2 to M1 further enhances phagocytosis and activates the tumor immune microenvironment. CTLs proliferate and infiltrate into the tumors to exert cytotoxic effects on tumor cells. PD-L1 upregulation in tumor cells also sensitizes their blockade by αPD-L1 to invigorate T cells. The figure was created with BioRender.com.

OxPt/SN38 causes CRT exposure on the cancer cell surface and release of HMGB-1 and other DAMPs to recruit antigen-presenting cells (APCs) into the tumors (Figure 6). By cross-linking DNAs and inhibiting topoisomerase I simultaneously, OxPt/SN38 causes severe DNA double-strand breaks (DSBs) to activate IRF1 and upregulate PD-L1 expression on tumor cells and DCs. OxPt/SN38 and αPD-L1 combination treatment increases tumor-infiltrating lymphocytes and antigen-specific IFN-γ-producing T cells in the spleens and repolarizes M2 macrophages into M1 macrophages to activate the tumor immune microenvironment. These mechanistic details support the synergistic combination of OxPt/SN38 and αPD-L1 in eliciting potent antitumor immunity.

In summary, we have elucidated the two-stage release of SN38 from OxPt/SN38 to enhance drug deposition in tumors. With SN38-TMS as the circulating species in the blood and by preferentially hydrolyzing SN38-TMS to SN38 in acidic TMEs, OxPt/SN38 reduces SN38 blood exposure and increases SN38 tumor exposure. OxPt/SN38 inhibits tumor growth on subcutaneous, spontaneous, and metastatic tumor models and exhibits strong synergy with αPD-L1 to eradicate subcutaneous colorectal and pancreatic tumors and greatly inhibits tumor growth and invasion in a spontaneous prostate cancer model and a liver metastasis model of colorectal cancer without causing side effects. The two-stage active drug release strategy can be combined with other innovative nanotechnology platforms to design potent nanomedicines for effective cancer therapy.

## Methods

### Chemicals, Cell Lines, and Animals

All starting chemicals were purchased from Sigma-Aldrich and Fisher Scientific (USA) unless otherwise noted and used without further purification. 1,2-Distearoyl-sn-glycero-3-phosphoethanolamine-*N*-[amino(polyethylene-glycol)2000] (DSPE-PEG_2k_), 1,2-dioleyl-sn-glycero-3-phosphocholine (DOPC), 1,2-dioleoyl-sn-glycero-3-phosphate (DOPA), and cholesterol were purchased from Avanti Polar Lipids (USA). OxPt/SN38 was synthesized and characterized as previously described.^32^

Murine colon carcinoma cells CT26 and MC38 were obtained from the American Type Culture Collection (Rockville, MD, USA). Dr. Hidayatullah G. Munshi at the Feinberg School of Medicine at Northwestern University originally provided murine pancreatic cancer cell line KPC. The MC38-OVA-luciferase cell line was obtained from Dr. Ralph Weichselbaum’s lab. The cells were cultured in RPMI-1640 medium (Gibco, Grand Island, NY, USA) or Dulbecco’s Modified Eagle’s Medium (DMEM) and then supplemented with 100 U/mL penicillin G sodium, 100 g/mL streptomycin sulfate, and 10% fetal bovine serum in a humidified atmosphere containing 5% CO_2_ at 37 °C.

BALB/c female mice (6 weeks, 18–22 g), C57BL/6 female mice (6 weeks, 18–22 g), and Sprague–Dawley female rats (6 weeks, 160–200 g) were purchased from Harlan Laboratories, Inc. (USA). The study protocol was reviewed and approved by the Institutional Animal Care and Use Committee (IACUC) at the University of Chicago.

### SN38 Release from OxPt/SN38

Twenty μM IRI or OxPt/SN38 was incubated in 10 unit/mL esterase (pH = 7.4 PBS, 37 °C) for 8 h or in pH = 4.7, 5.5, and 6.5 in PBS at 37 °C for 72 h. For IRI, 300 μL of methanol was added to 100 μL of the aliquot to precipitate proteins. After centrifugation (14000*g*, 5 min), the supernatant from the suspension was analyzed for IRI by LC-MS. For OxPt/SN38, 100 μL of aliquot was added with 5 μL of 20% Triton X-100 aqueous solution, 100 μL of saturated NaCl solution, and 100 μL of ethyl acetate. The solution mixture was vortexed for 30 s and then centrifuged as above. LC-MS was used to analyze the organic layer.

Conversion of SN38-TMS to SN38 was examined by incubating 20 μM SN38-TMS in pH = 7.4 or pH = 4.7 PBS with 3% DMSO at 37 °C for 24 h. A 100 μL portion of the aliquot was added with 5 μL of 20% Triton X-100 aqueous solution, 100 μL of saturated NaCl solution, and 100 μL of ethyl acetate. The mixture was vortexed for 30 s and then centrifuged as above. LC-MS was used to analyze the organic layer.

### *In Vitro* Cytotoxicity

In 96-well plates were seeded 2 × 10^3^ cells/well of MC38 or CT26 tumor cells. After 24 h, IRI, Chol-SN38, SN38, SN38-TMS, ZnP/SN38, OxPt, OxPt NCP, or OxPt/SN38 was added into the media at different concentrations for 48 h treatment. A 3-(4,5-dimethylthiazol-2-yl)-5-(3-carboxymethoxyphenyl)-2-(4-sulfophenyl)-2*H*-tetrazolium (MTS, Promega, Madison, WI) assay was used to determine cell viability.

### Pharmacokinetics and Biodistribution Analysis

Sprague–Dawley rats were i.v. injected with OxPt plus IRI or OxPt/SN38 at a dose of 2.0 mg OxPt/kg and 3.6 mg SN38/kg. The blood was collected at 5 min, 30 min, and 1, 3, 5, 8, 24, and 48 h postinjection and centrifuged at 604*g* for 10 min to harvest plasmas. For Pt detection, 20 μL of plasma was digested with concentrated HNO_3_ for more than 1 day and analyzed by ICP-MS. For the OxPt plus IRI study, a 20 μL aliquot was diluted with 80 μL of PBS and then added to 300 μL of methanol to precipitate the proteins. The mixture was then centrifuged at 14000*g* for 5 min. The supernatant was analyzed for IRI and SN38 by LC-MS. For the OxPt/SN38 study, 20 μL of plasma was added to 100 μL of saturated NaCl solution and 5 μL of 20% Triton X-100, and then the mixture was extracted with 100 μL of ethyl acetate, followed by 10 min of centrifugation at 6708*g*. The organic layer was analyzed for Chol-SN38, SN38-TMS, and SN38 by LC-MS.

The right flanks of C57Bl/6 mice were subcutaneously injected with 1 × 10^6^ MC38 cells. When the tumors reached ∼100 mm^3^, the mice were i.v. injected with OxPt plus IRI or OxPt/SN38 at a dose of 3.5 mg/kg OxPt and 6.2 mg/kg SN38 equiv. The spleens, lungs, kidneys, livers, and tumors were collected at 1, 3, 8, 24, 48, and 72 h after injection. ICP-MS was used to quantify Pt, while LC-MS was used to quantify IRI, Chol-SN38, and SN38.

### DNA Double-Strand Breaks

DNA double-strand breaks were determined by phosphorylated γ-H2AX detection. For CLSM, MC38 cells were cultured in a 6-well plate at 5 × 10^5^ cells per well overnight and incubated with OxPt/SN38, OxPt plus IRI, or PBS at 5 μM OxPt and 9 μM SN38. Twenty-four h later, cells were fixed with 4% paraformaldehyde (pH = 7.2) at RT for 10 min, washed with PBS, and blocked and permeabilized by 5% FBS and 0.3% Triton-X in PBS at room temperature for 1 h. The cells were incubated with primary antibodies in 1% BSA and 0.3% Triton-X in PBS at 4 °C overnight (phosphohistone H2A.X (Ser139) (20E3) rabbit mAb #9718, 1:400). The cells were then washed with PBS and incubated with secondary antibodies in 1% BSA and 0.3% Triton-X in DPBS at room temperature for 1 h (antirabbit IgG (H+L), F(ab′)2 fragment (Alexa Fluor 647 conjugate) #4414, 1:1000). After washing with PBS, the cover slips were mounted on glass slides with ProLong Antifade Mountant with DAPI (ThermoFisher Scientific), cured at 4 °C overnight, sealed by nail polish, and observed on a Leica SP8 confocal microscope.

For flow cytometry, MC38 cells were cultured in a 6-well plate at 5 × 10^5^ per well overnight and incubated with PBS, OxPt, IRI, OxPt plus IRI, ZnP/SN38, OxPt NCP, or OxPt/SN38 (5 μM OxPt, 9 μM SN38 equiv; the dose was used for all *in vitro* studies). Twenty-four h later, cells were harvested from the plates, transferred to Eppendorf tubes, fixed with 4% paraformaldehyde (pH = 7.2) at room temperature for 10 min, washed with PBS, and blocked and permeabilized by 5% FBS and 0.3% Triton-X in PBS at room temperature for 1 h. The cells were incubated with primary antibodies in 1% BSA and 0.3% Triton-X in PBS at 4 °C overnight (phosphohistone H2A.X (Ser139) (20E3) rabbit mAb #9718, 1:400). The cells were then washed with PBS and incubated with secondary antibodies in 1% BSA and 0.3% Triton-X in DPBS at room temperature for 1 h (antirabbit IgG (H+L), F(ab′)2 fragment (Alexa Fluor 647 conjugate) #4414, 1:1000). After washing with PBS, the cells were resuspended with FACS buffer and analyzed on an LSR Fortessa 4-15 flow cytometer.

### Western Blot

All antibodies used in Western blot experiments were purchased from Cell Signaling Technology, except for anti-PD-L1 antibody (Abcam). All buffers, assays, and XCell SureLock Mini-Cell were obtained from ThermoFisher Scientific. The mini trans-blot electrophoretic transfer cell was purchased from Bio-Rad, and the FluorChem R system was obtained from ProteinSimple. Cells were lysed by RIPA buffer with protease and phosphatase inhibitor cocktail following the manufacturer’s specifications. The proteins in the supernatant were collected by centrifugation at 14000*g* and the concentrations were measured and normalized by a BCA assay. The proteins were denatured and reduced by NuPAGE LDS sample buffer with 50 mM DTT and then heated to 70 °C for 10 min. Ten to 20 μg of samples were loaded on 4–12% NuPAGE Bis-Tris gel for electrophoresis on a XCell SureLock Mini-Cell (200 V, 35–50 min) and electr-transferred to a PVDF membrane (200 mA, 90 min) on a mini trans-blot electrophoretic transfer cell. The membrane was blocked by TBST with 5% nonfat dry milk at room temperature for 1 h and incubated with primary antibody solution in TBST with 5% BSA at 4 °C overnight (Phosphohistone H2A.X (Ser139) (20E3) rabbit mAb #9718, 1:2000; IRF-1 (D5E4) XP rabbit mAb #8478 1:1000; anti-PD-L1 antibody (ab233482), 1:1000). The membrane was washed with TBST and incubated with secondary antibody with HRP conjugate in TBST with 5% BSA at room temperature for 1 h (antirabbit IgG, HRP-linked antibody #7074, 1:2000–5000; antimouse IgG, HRP-linked antibody #7076, 1:5000). The membrane was again washed with TBST, and a Pierce ECL Western blotting substrate was added.

### PD-L1 Expression

MC38 cells were cultured in a 6-well plate at 5 × 10^5^ cells per well overnight and incubated with PBS, OxPt, IRI, OxPt plus IRI, ZnP/SN38, OxPt NCP, or OxPt/SN38. Twenty-four h later, cells were harvested from the plate, transferred to Eppendorf tubes, and incubated with anti-CD16/32 (clone 93; eBiosciences, 1:100) to reduce nonspecific binding to FcRs. Cells were further stained with the APC antimouse CD274 (B7-H1, PD-L1) antibody (clone:10F.9G2; Biolegend, 1:100) on ice for 1 h. After washing with PBS, the cells were resuspended with FACS buffer and analyzed on an LSR Fortessa 4–15 flow cytometer.

### ROS Generation

For CLSM, MC38 cells were treated with OxPt/SN38, OxPt plus IRI, or PBS for 24 h and incubated with 10 μM H2DCFDA (Thermo Fisher, USA) for another 1 h. The cells were fixed with 4% paraformaldehyde (pH = 7.2) at room temperature for 10 min and washed with PBS. The cover slips were mounted on glass slides with ProLong glass antifade mountant with DAPI, cured at 4 °C overnight, sealed by nail polish, and observed on a Leica SP8 confocal microscope.

For flow cytometry, MC38 cells were treated with PBS, OxPt, IRI, OxPt plus IRI, ZnP/SN38, OxPt NCP, or OxPt/SN38 for 24 h and incubated with 10 μM H2DCFDA (Thermo Fisher, USA) for an additional 1 h. The cells were collected, washed twice with ice-cold PBS, and analyzed by flow cytometry.

### CRT Exposure

For cell surface CRT detection, MC38 cells were seeded on 10 mm^2^ glass cover slips placed in 6-well plates at a density of 2 × 10^5^ cells per well and cultured with PBS, OxPt plus IRI, or OxPt/SN38 for 24 h. After treatments, cells were washed three times with PBS and incubated with Alexa Fluor 488-CRT antibody (Enzo cat # ADI-SPA-601–488-F, diluted 1:100) for 2 h. After washing with PBS, the cover slips were mounted on glass slides with ProLong glass antifade mountant with DAPI, cured at 4 °C overnight, sealed by nail polish, and observed on a Leica SP8 confocal microscope.

Two ×10^5^ cells/well of MC38 cells were seeded in 6-well plates and then cultured with PBS, OxPt, IRI, OxPt plus IRI, ZnP/SN38, OxPt NCP, or OxPt/SN38 for 24 h. The cells were harvested, incubated with Alexa Fluor 488-CRT antibody for 2 h, stained with PI, and analyzed by flow cytometry to identify CRT exposure. The fluorescence intensity of stained cells was gated on PI– cells.

For *in vivo* CRT staining, the tumors were harvested and frozen with OCT compound (Fisher Healthcare) at −80 °C. The blocks were sectioned, fixed with acetone at −20 °C for 10 min, washed with TBST to remove OCT, blocked by 5% FBS in PBS, incubated with Alexa Fluor 488-CRT antibody (diluted 1:100) at a 4 °C wet chamber overnight, and observed by a Leica SP8 confocal microscope.

### HMGB-1 Release

Two ×10^5^ cells/well of MC38 cells were seeded in 6-well plates and then cultured with PBS, OxPt, IRI, OxPt plus IRI, ZnP/SN38, OxPt NCP, or OxPt/SN38 for 24 h. The medium was collected to detect HMGB-1 release by ELISA (Chondrex, Redmond, WA).

### *In Vivo* Anticancer Efficacy

The right flank regions of 6-week BALB/c (for CT26) or C57Bl/6 (for MC38 and KPC) wild-type mice were subcutaneously injected with 2 × 10^6^ cells CT26, MC38 or KPC cells, respectively. Seven days later, mice were i.v. injected with PBS, OxPt plus IRI, OxPt NCP, ZnP/SN38, or OxPt/SN38 at a dose of 3.5 mg/kg OxPt and 6.2 mg/kg SN38 (this dose was used in all other *in vivo* studies) once every 3 days for 6 doses (for CT26 and KPC) or 8 doses (for MC38). A 75 μg portion of αPD-L1 (Clone: 10F.9G2, 564 Catalog No. BE0101, BioXCell) was i.p. injected in the same schedule. Tumors were measured with a digital caliper, where their volumes were calculated as ((width)^2^ × length)/2. Tumor growth inhibition (TGI) was defined as
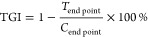
where *T*_end point_ and *C*_end point_ refer to the tumor sizes of treated mice and PBS control, respectively.

### Hematological Analysis

Blood samples were collected from C57BL/6 mice i.v. injected with PBS, free OxPt, free IRI, OxPt plus IRI, OxPt NCP, ZnP/SN38, or OxPt/SN38 once every 3 days for eight doses and analyzed using a COULTER Ac•T 5diff CP hematology analyzer (Beckman Coulter).

### Spontaneous TRAMP Model

B6 TRAMP mice (C57BL/6-Tg(TRAMP)8247Ng/J) breeding pairs from the Jackson Laboratory were housed and bred as instructed. The TRAMP offspring strain mice were selected by genotyping. Twenty-four week-old TRAMP mice were i.v. injected with PBS, OxPt plus IRI plus αPD-L1, OxPt/SN38, or OxPt/SN38 plus αPD-L1 once every 3 days for 10 doses. A 75 μg portion of αPD-L1 was i.p. injected in the same schedule. Thirty days after the first injection, the prostates and seminal vehicles (genitourinary tracts) were dissected and normalized against the mouse body weight.

### Liver Metastasis Model

Mice were placed on a heating pad and anesthetized with 3% isoflurane (induction) and 2% isoflurane (maintenance). The abdomen or tail was sprayed with 70% ethanol before surgery or injection. While the mice were placed in the right lateral recumbent position, a 1 cm incision was made in the left upper abdominal wall of each mouse. A 1 cm incision was made in the peritoneum to expose the spleen. The spleen was gently injected with 5 × 10^6^ MC38-OVA-luciferase cells in 50 μL of PBS. The insertion site of the needle was cauterized and sealed with GEMINI cautery (Braintree Scientific, Inc.) to reduce bleeding. Five min after injection, splenectomy was performed using GEMINI cautery. The abdominal incision was closed in two layers with 5-0 polydioxanone absorbable thread (AD Surgical, Sunnyvale, CA, USA). Mice were anesthetized by the XGI-8 Gas Anesthesia system (2% isoflurane; Xenogen, Alameda, CA, USA). The In Vivo Imaging System (IVIS) Lumina XR (Xenogen) was used to measure fluorescence intensity. Living Image Ver.4.5 (Xenogen) image software was used to acquire an image sequence. The region of interest was defined in the upper abdominal area to obtain the photon flux data.

### Hematoxylin and Eosin (H&E) and IHC Staining

Healthy C57BL/6 mice (female, *n* = 6) were treated with OxPt plus IRI or OxPt/SN38 once every 3 days for eight doses. At day 22 after the first dose, mice were euthanized, and gross necropsies were performed. Target tissues were collected, fixed with 4% paraformaldehyde, embedded in paraffin, and cut into sections for hematoxylin and eosin (H&E) staining before histopathological examination with a Pannoramic MIDI II Digital Slide Scanner.

### Immune Cell Profiling

MC38 tumor-bearing C57BL/6 mice were dosed with PBS, OxPt plus IRI plus αPD-L1, OxPt/SN38, or OxPt/SN38 plus αPD-L1 Q3D for two or five doses, and the tumors were harvested on day 4 or day 13, respectively, for immune cell profiling by flow cytometry. The tumors were digested by RPMI-1640 with 10% FBS, 1 mg/mL of collagenase I (Gibco), 250 μg/mL of collagenase IV (Gibco), and 50 μg/mL of DNase I (Sigma-Aldrich) at 37 °C for 45 min. The digests were gently ground and filtered through sterile cell strainers (40 μm, Corning) to produce single-cell suspensions. The cells were washed with ice-cold FACS buffer and stained first with a LIVE/DEAD fixable yellow dead cell stain kit (ThermoFisher Scientific, 1:1000). The cells were then washed with FACS buffer, blocked by anti-CD16/32 antibody (clone 93, 1:100) at 4 °C for 30 min, and stained with the following fluorochrome conjugated rat antimouse antibodies 1:200 at 4 °C for 45 min: PD-L1-APC (10F.9G2) CD45-BV421 (30-F11), CD11b-FITC (M1/70), F4/80-PerCP/Cy5.5 (BM8), Gr-1-PE (RB6-8C5), CD86-APC (GL1), CD206-PE/Cy7 (C068C2), CD11c-PE/Cy5.5 (N418), PD-L1-APC and CD3ε-PE/eFluor610 (145-2C11), CD4-APC/H7 (GK1.5), CD8α-PerCP/eFluor710 (53–6.7). CD45-BV421 was obtained from BD Bioscience. CD206-PE/Cy7 was obtained from BioLegend. Other antibodies were obtained from eBioscience. The cells were finally washed and resuspended in FACS buffer and analyzed on an LSR Fortessa 4-15 flow cytometer. Representative gating strategies are given in Figures S12 and S13.

### IFN-γ ELISPOT Assay

A Multiscreen HTS-IP plate (Millipore Sigma) was activated by 70% ethanol, washed with PBS, coated with antimouse IFN-γ capture antibody (BD Biosciences) at 37 °C for 8 h, and blocked with sterile 1% BSA in PBS at RT for 2 h. The spleens were harvested from the treated MC38 tumor-bearing C57BL/6 mice and then gently ground and filtered through sterile cell strainers to afford single-cell suspensions. Red blood cells were then lysed by sterile ACK buffer (Corning), and splenocytes were counted and seeded in the plate at a density of 2 × 10^5^ cells/well in RPMI-1640 full medium (6 mice each treatment group and each mouse with 3 replicates). MC38 tumor associated KSPWFTTL (KSP) peptide was added to each well at a concentration of 10 μg/mL except for negative control wells. The splenocytes in positive control wells were directly stimulated with antimouse CD3ε (145-2C11) and antimouse CD28 (37.51) antibody (eBioscience, 1:1000). The splenocytes were incubated at 37 °C for 48 h, and culture media were discarded. The plates were then washed and incubated with biotinylated anti-IFN-γ detection antibody, streptavidin-HRP conjugate, and AEC substrate following the manufacturer’s specification (BD Biosciences). The plate was air-dried and analyzed by a CTL ImmunoSpot S6 Analyzer.

### Statistical Analysis

To ensure an appropriate statistical ANOVA analysis for efficacy studies, *n* ≥ 5 was used for each group. Student’s *t* tests were used to determine if the variance between groups is similar. OriginPro (OriginLab Corp.) was used to perform a statistical analysis. The survival curves were analyzed by a Kaplan–Meier survival analysis with the log-rank (Mantel–Cox) test. Statistical significance was calculated using two-tailed Student’s *t* tests and defined as *p < 0.05, **p < 0.01, ***p < 0.001, ****p < 0.0001. Animal experiments were not performed in a blinded fashion and are represented as mean ± SD.

## Data Availability

The authors declare that all the data supporting the findings of this study are available within the article and its Supporting Information files or from the corresponding author upon reasonable request.

## Supporting Information Available

Synthesis and characterization of Chol-SN38-OH and OxPt/SN38, *in vitro* studies including cytotoxicity, double-strand break, PD-L1 expression, ROS detection, and ICD generation, and *in vivo* studies including antitumor efficacy, safety evaluation, and leukocyte profiling (PDF)
