# Supplementary material for: Two-Stage SN38 Release from a Core–Shell Nanoparticle Enhances Tumor Deposition and Antitumor Efficacy for Synergistic Combination with Immune Checkpoint Blockade
Source: ACS Nano. 2022 Nov 16;16(12):21417–30. doi: 10.1021/acsnano.2c09788 (PMC9798857; doi:10.1021/acsnano.2c09788)
Supplement: Supplementary file 1 — nn2c09788_si_001.pdf [file nn2c09788_si_001.pdf]

Supporting Information for

Two-Stage SN38 Release from A Core-Shell Nanoparticle Enhances Tumor Deposition and Antitumor Efficacy for Synergistic Combination with Immune Checkpoint Blockade

Xiaomin Jiang,[1] Morten Lee,[1] Junjie Xia,[1] Taokun Luo,[1] Jianqiao Liu,[1] Megan Rodriguez,[1] Wenbin Lin[1,2,*]

[1]Department of Chemistry, The University of Chicago, 929 E 57th St, Chicago, IL 60637, USA.

[2]Department of Radiation and Cellular Oncology and Ludwig Center for Metastasis Research, The University of Chicago, 5758, S Maryland Ave, Chicago, IL 60637, USA.

[*]Corresponding author. E-mail: wenbinlin@uchicago.edu

Synthesis and characterization of 7-ethyl-10-(((((cholest-5-en-3-oxy)-4-oxobutanoyl)oxy)methoxy)carbonyl)oxyl- camptothecin (Chol-SN38-OH)

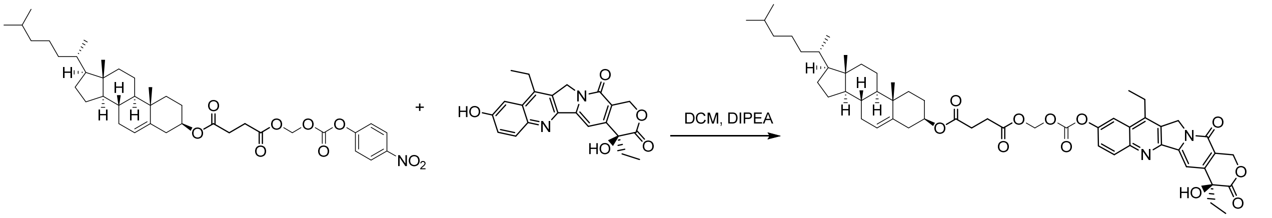

7 g (10 mmol) cholest-5-en-3-ol ((((4-nitrophenoxy)carbonyl)oxy)methyl) succinate and 5 g SN38 (12.7 mmol, 1.27 eq) were dissolved in 250 mL anhydrous dichloromethane. 10 mL (60 mmol, 6 eq) DIPEA was then added, and the resultant solution was stirred at room temperature under nitrogen protection for 24 h. The solution was diluted with 500 mL dichloromethane and washed with saturated $NaHCO_3$ aqueous solution three times, 1M HCl solution once, and then saturated NaCl solution once. The organic phase was dried over anhydrous Na_2SO_4 for 2 h and concentrated by rotary evaporation. The product was purified by column chromatography on silica using 15:1 dichloromethane: ethyl acetate (V/V) as eluent. Yield: 8 g (8 mmol, 80%). ^{1}H NMR (500 MHz, Chloroform-*d*) δ 8.28 (d, *J* = 9.2 Hz, 1H), 7.96 (d, *J* = 2.6 Hz, 1H), 7.68 (dd, *J* = 9.2, 2.6 Hz, 1H), 7.52 (s, 1H), 5.93 (s, 2H), 5.68 (d, *J* = 16.6 Hz, 1H), 5.34 – 5.19 (m, 5H), 4.61 (dt, *J* = 7.8, 2.2 Hz, 1H), 3.16 (d, *J* = 7.7 Hz, 2H), 2.79 – 2.73 (m, 2H), 2.70 – 2.63 (m, 2H), 2.30 (d, *J* = 7.7 Hz, 2H), 2.02 – 1.73 (m, 8H), 1.63 – 0.74 (m, 38H), 0.63 (s, 3H). HRMS: m/z=935.5024 (expected 935.4980 for $[M+H]^+$).

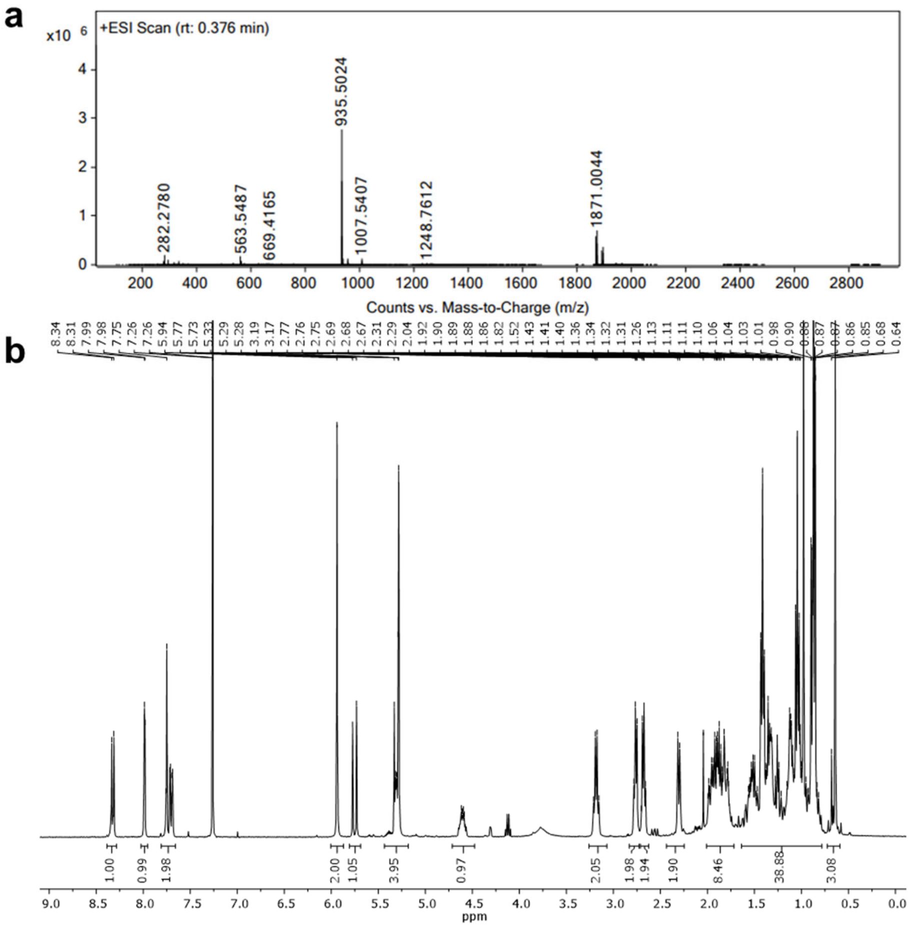

Figure S1 (a) HRMS of Chol-SN38-OH. (b) ^{1}H NMR spectrum of Chol-SN38-OH in $CDCl_3$.

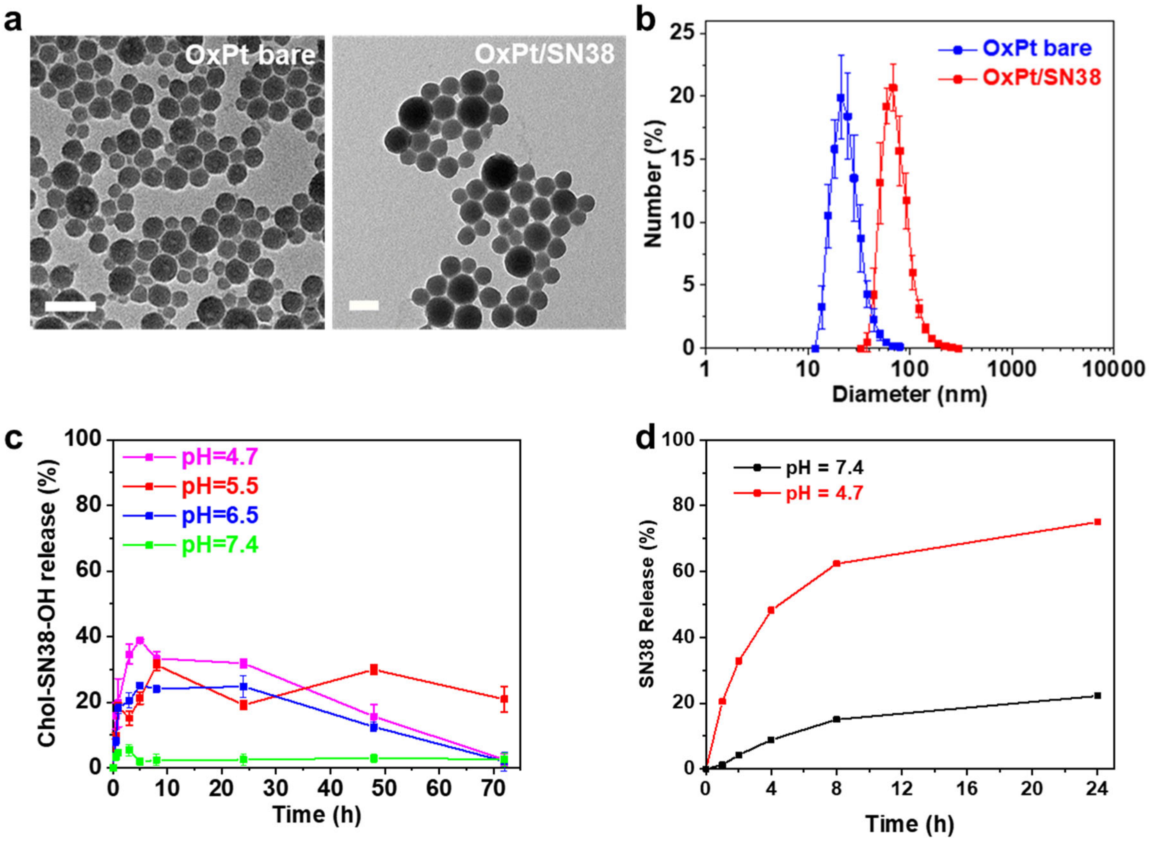

Figure S2 (a) TEM image of OxPt-bare and OxPt/SN38. (b) Number-average diameter of OxPt-bare and OxPt/SN38. (c) Time-dependent release of the intermediate Chol-SN38-OH from OxPt/SN38 in pH=4.7, 5.5, 6.5 and 7.4 PBS at 37 ºC. (d) Conversion of SN38-TMS to SN38 in different pH conditions. PBS solutions of 20 ppm SN38-TMS were prepared in different pH conditions (pH = 4.7 and 7.4) and the amounts of SN38-TMS and SN38 were measured by LC-MS after incubation in 37 °C.

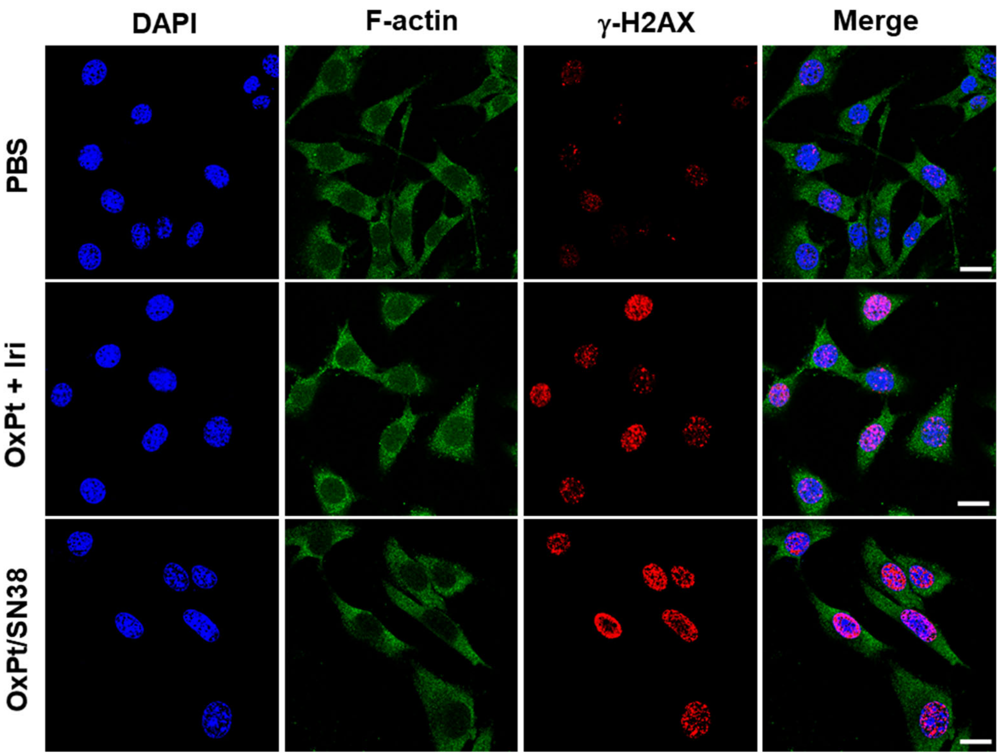

Figure S3 CLSM images showing DNA DSBs 24 hours after treatment of MC38 cells with 5 μM OxPt plus 9 μM IRI or OxPt/SN38 at the same equivalent concentrations, scale bar = 20 μm.

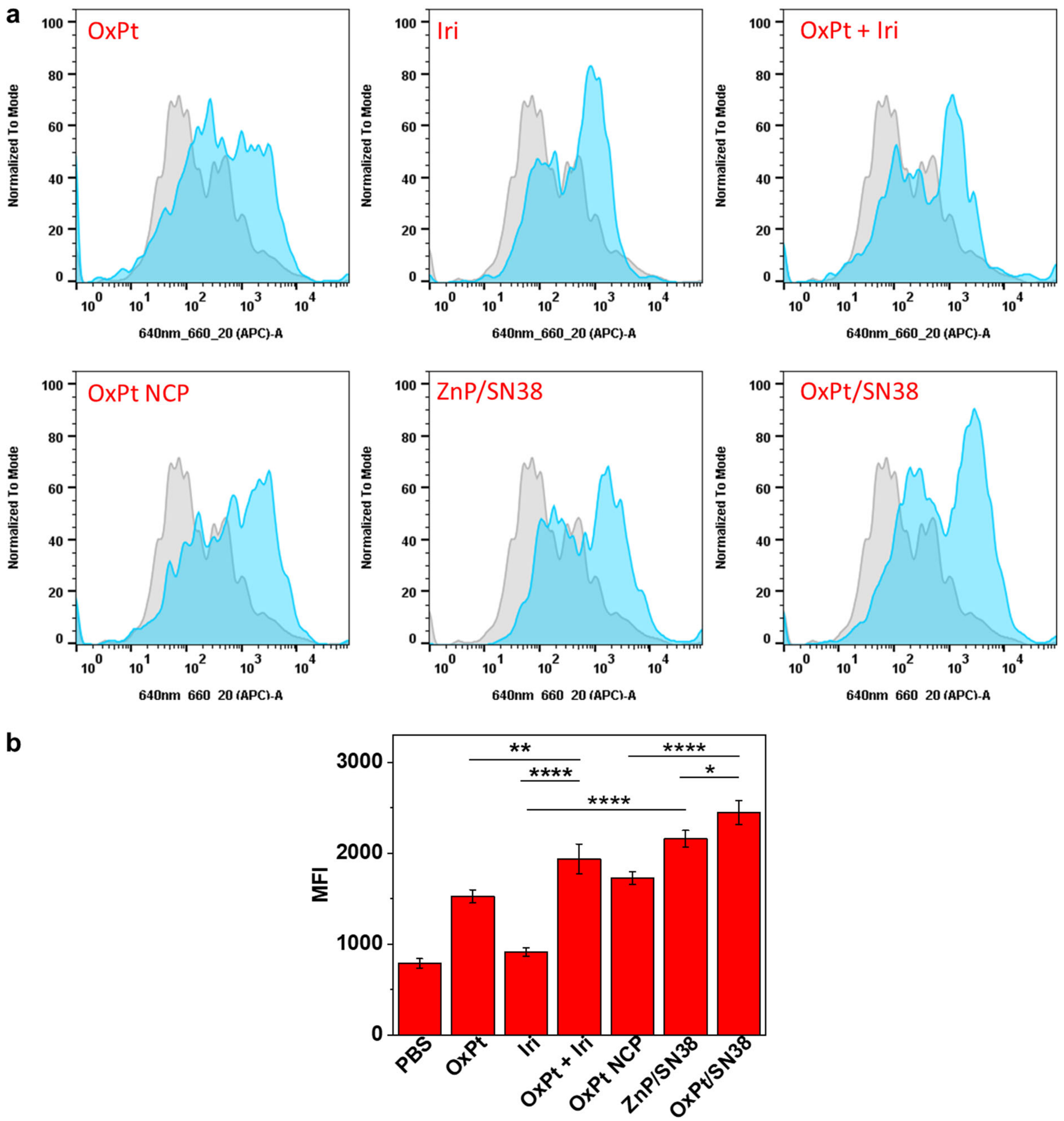

Figure S4 Flow cytometry (a) and statistical analysis results (b) of γ-H2AX of MC38 cells after treatment with OxPt, IRI, OxPt plus IRI, OxPt NCP, ZnP/SN38, or OxPt/SN38 for 24 hours. OxPt dose was 5 μM and IRI dose was 9 μM. Grey histograms represent signals from the PBS group.

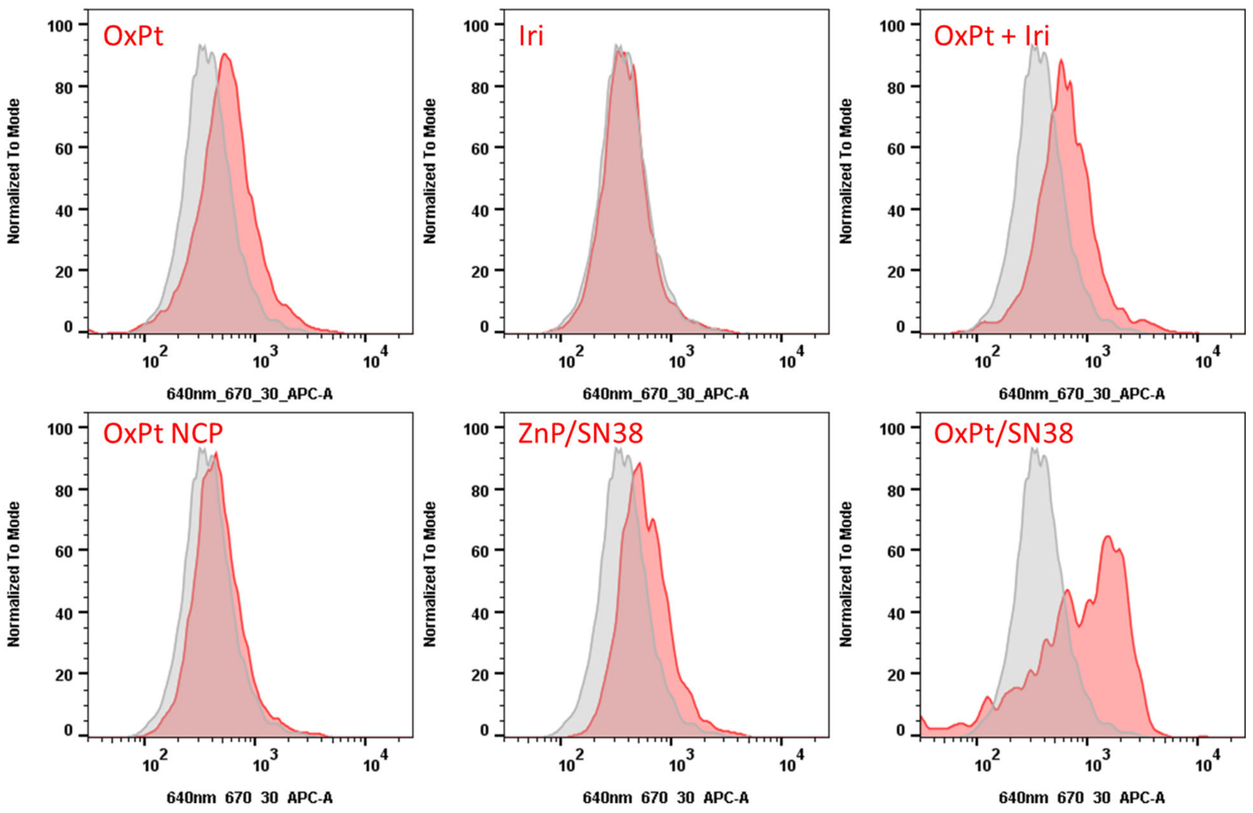

Figure S5 Flow cytometry results showing PD-L1 expression on MC38 cells after treatment with OxPt, IRI, OxPt plus IRI, OxPt NCP, ZnP/SN38, or OxPt/SN38 for 24 hours. OxPt dose was 5 μM and IRI dose was 9 μM. Grey histograms represent signals from the PBS group.

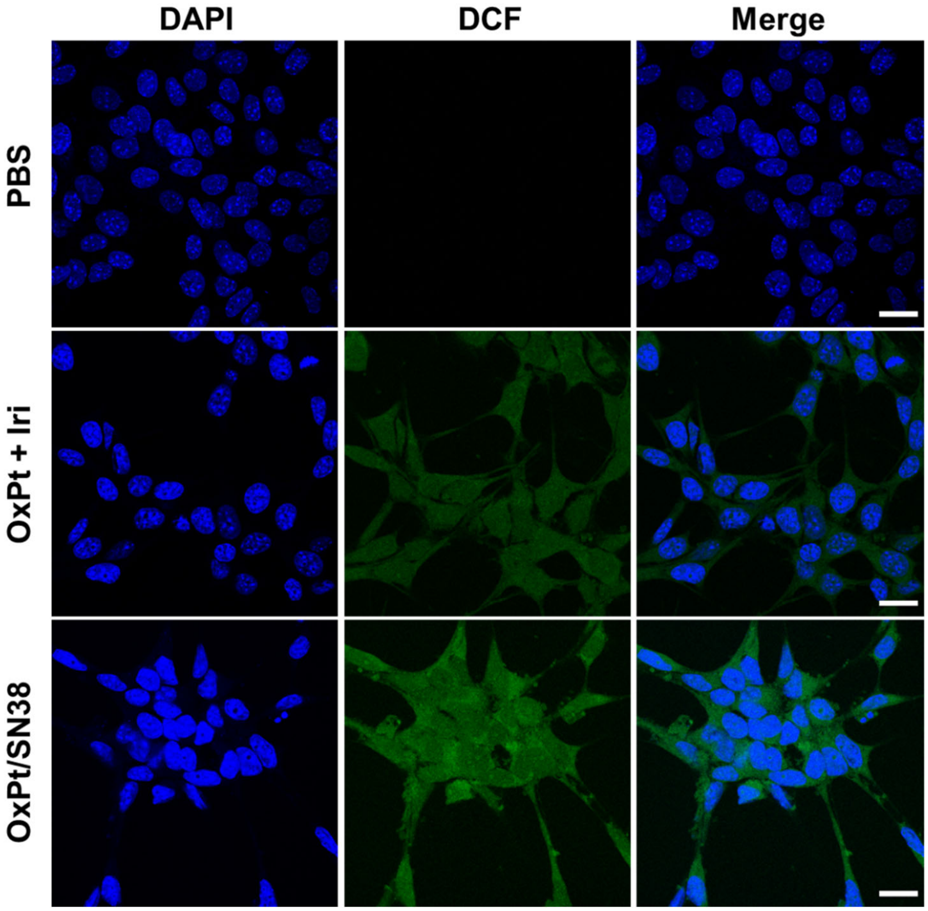

Figure S6 CLSM images showing DCF signals of MC38 cells after treatment with PBS, OxPt plus IRI, or OxPt/SN38 for 24 hours. OxPt dose was 5 μM and IRI dose was 9 μM., scale bar = 20 μm.

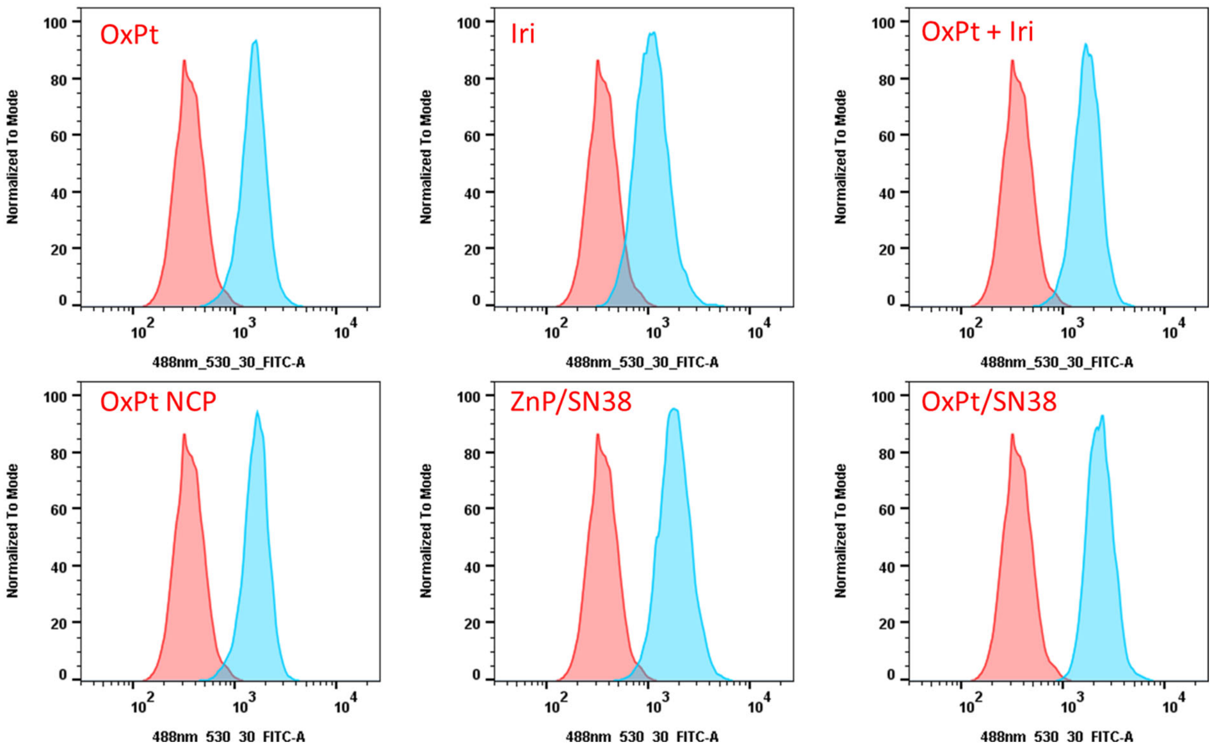

Figure S7 Flow cytometry (a) and statistical analysis results (b) of DCF signals of MC38 cells after treatment with OxPt, IRI, OxPt plus IRI, OxPt NCP, ZnP/SN38, or OxPt/SN38 for 24 hours. OxPt dose was 5 μM and IRI dose was 9 μM. Grey histograms represent signals from the PBS group.

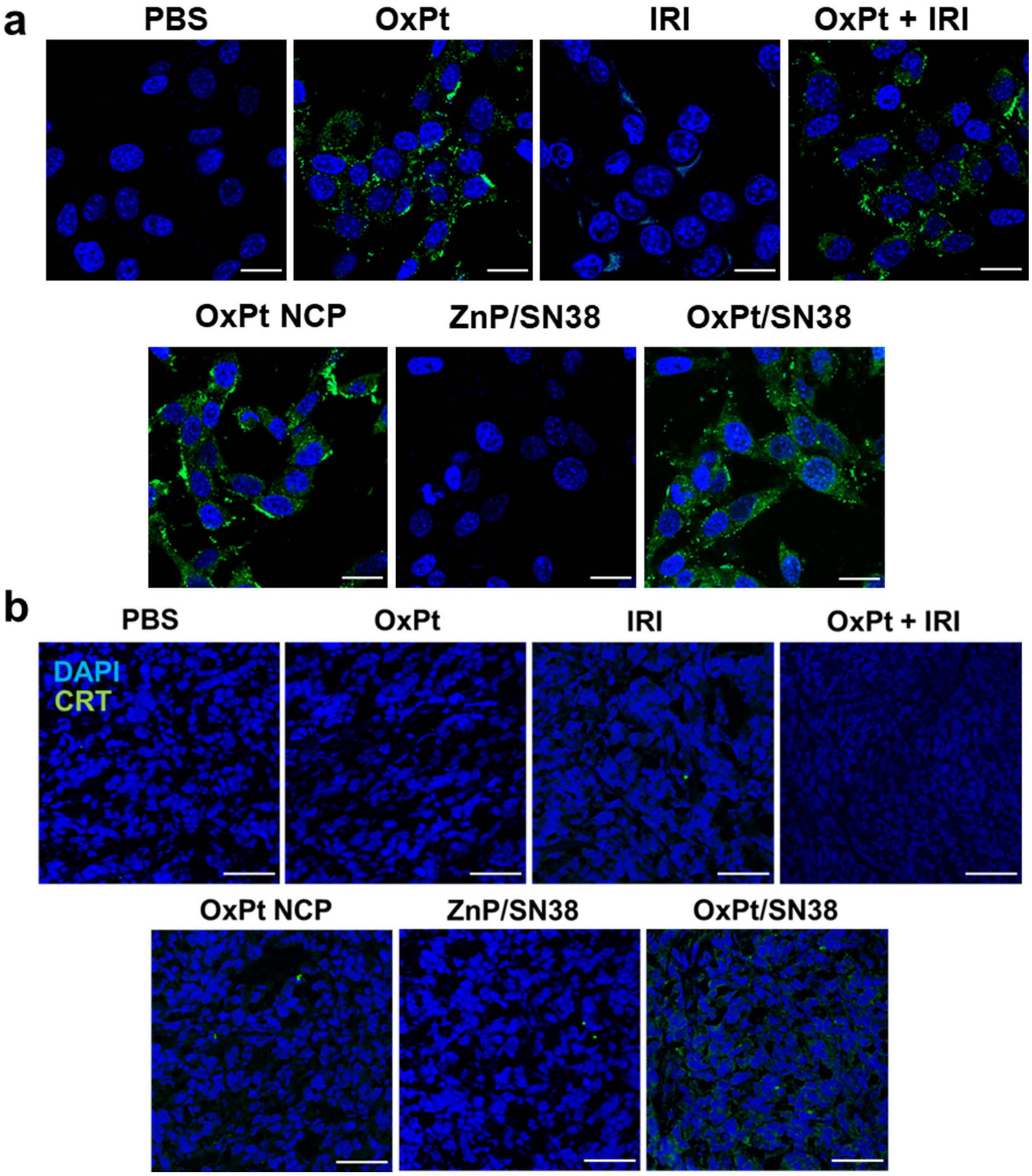

Figure S8 (a) CLSM images showing CRT signals on MC38 cells after treatment with PBS, OxPt, IRI, OxPt plus IRI, OxPt NCP, ZnP/SN38, or OxPt/SN38 for 24 hours. OxPt dose was 5 μM and IRI dose was 9 μM. Scale bar = 20 μm. (b) CRT staining of excised MC38 tumors after 8 doses of PBS, OxPt, IRI, OxPt plus IRI, OxPt NCP, ZnP/SN38, or OxPt/SN38. Scale bars are 50 μm.

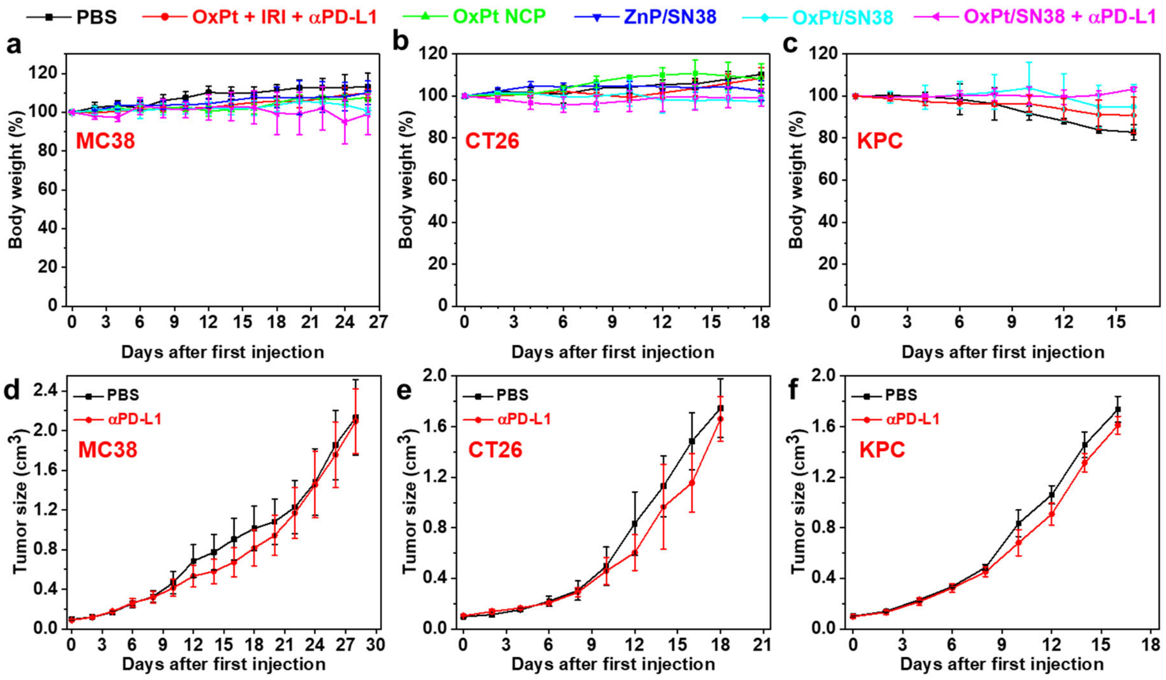

Figure S9 Body weights (a) of MC38 tumor-bearing C57BL/6 mice after indicated treatments, n = 6. Body weights (b) of CT26 tumor-bearing BALB/c mice after indicated treatments, n = 6. Body weights (c) of KPC tumor-bearing C57BL/6 mice after indicated treatments, n = 5. PBS, OxPt plus irinotecan, OxPt NCP, ZnP/SN38, and OxPt/SN38 were i.v. injected once every 3 days (Q3D) at doses of 3.5 mg/kg OxPt or equivalent and 6.2 mg/kg SN38 equivalent to the MC38 model for up to 8 doses and to CT26 and KPC models for up to 6 doses. 75 μg of αPD-L1 antibody (αPD-L1) was i.p. injected with the same dosing schedule. Growth curves of MC38 tumors in C57BL/6 mice (d), CT26 tumors in BALB/c mice (e), and KPC tumors in C57BL/6 mice (f) after repeated doses of 75 μg of αPD-L1. n = 6 for MC38 and CT26, n = 5 for KPC.

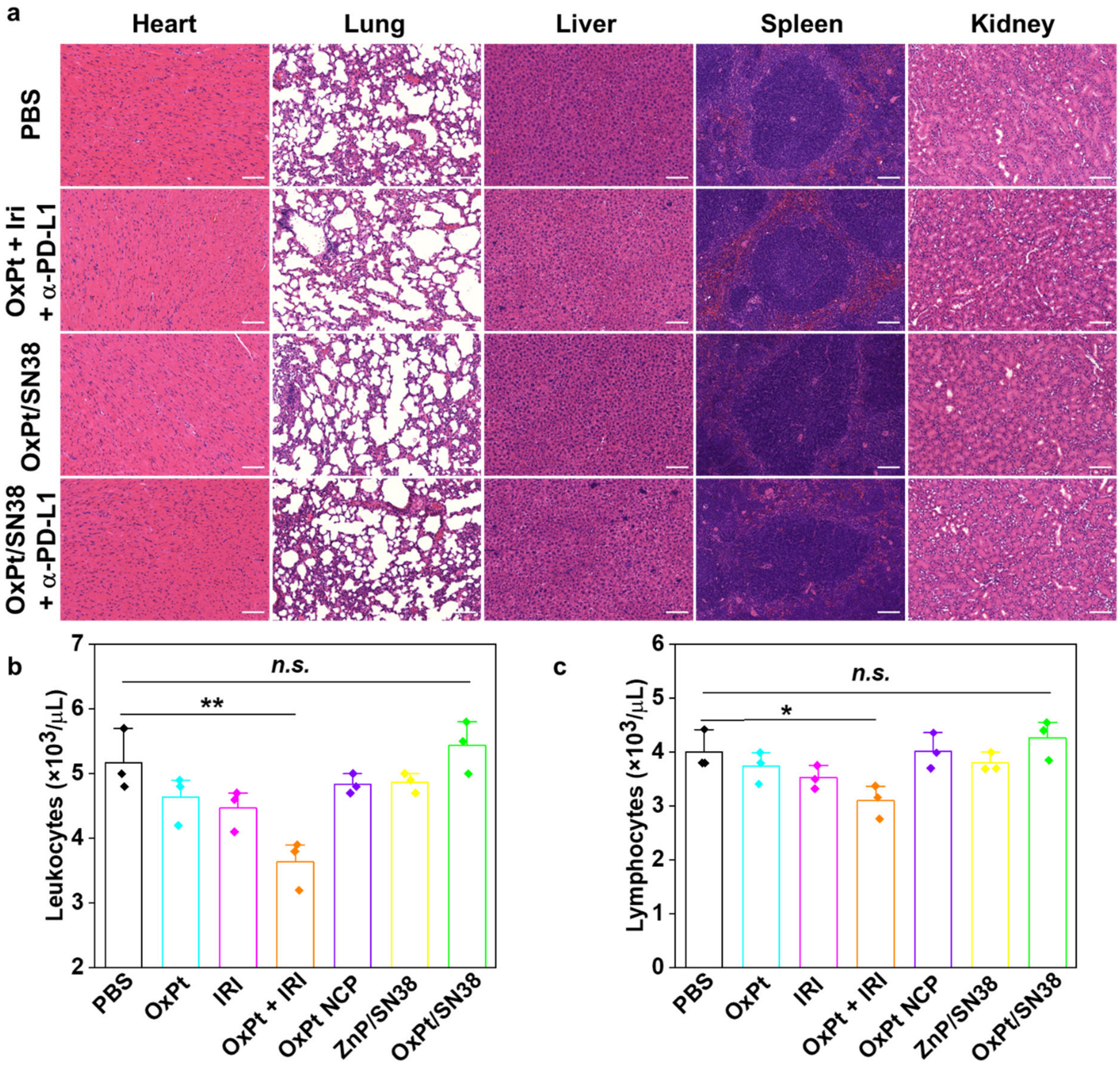

Figure S10 (a) The histologies of major organs of MC38 tumor-bearing C57BL/6 mice by hematoxylin and eosin (H&E) staining. The mice were i.v. injected with PBS, OxPt plus IRI, OxPt NCP, ZnP/SN38, and OxPt/SN38 once every 3 days at doses of 3.5 mg/kg OxPt or equivalent and 6.2 mg/kg SN38 equivalent for 8 doses. Scale bar = 100 μm. Leukocyte (b) and lymphocyte (c) counts of C57BL/6 mice after treatment with 8 i.v. injections of PBS, free OxPt, free IRI, OxPt plus IRI, OxPt NCP, ZnP/SN38 or OxPt/SN38 (Q3D).

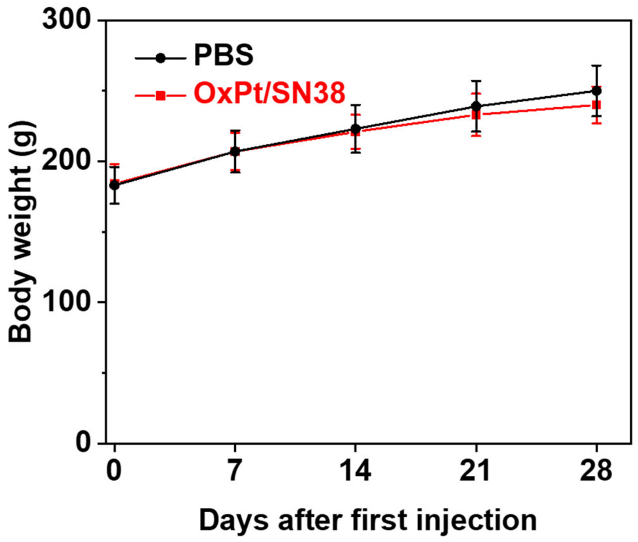

Figure S11 Body weights of female Sprague-Dawley rats i.v. injected with OxPt/SN38 at 4.0 mg/kg OxPt equivalent and 18.3 mg/kg Chol-SN38 once every week for 4 doses.

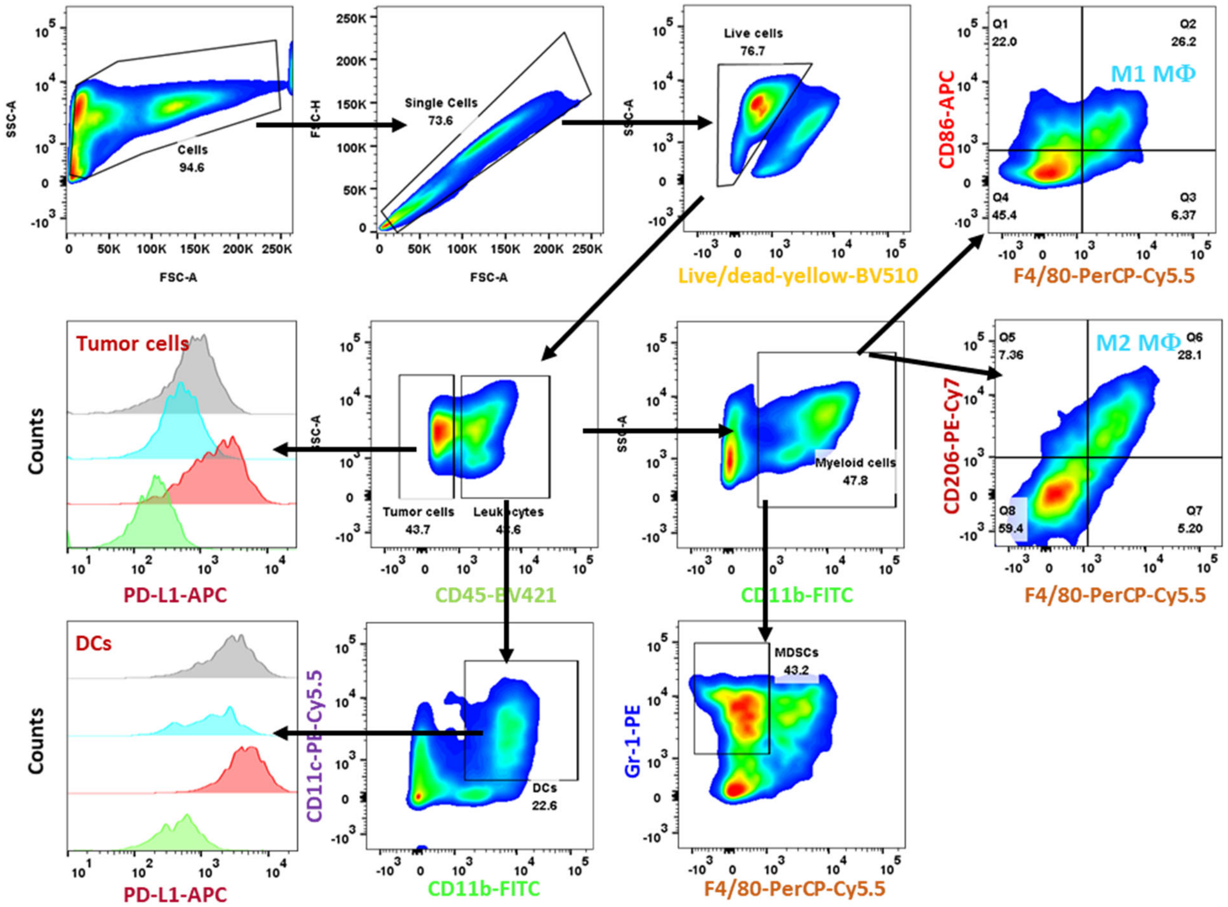

Figure S12 Gating strategies for leukocytes ($CD45^+$), myeloid cells ($CD45^+$ $CD11b^+$), MDSCs ($CD45^+$ $CD11b^+$ $GR\text{-}1^+$ $F4/80^-$), M1 macrophages (CD45+ $CD11b^+$ $F4/80^+$ $CD86^+$), M2 macrophages ($CD45^+$ $CD11b^+$ $F4/80^+$$CD206^+$), and DCs ($CD45^+$ $CD11b^+$ $CD11c^+$) in **Figure 5**.

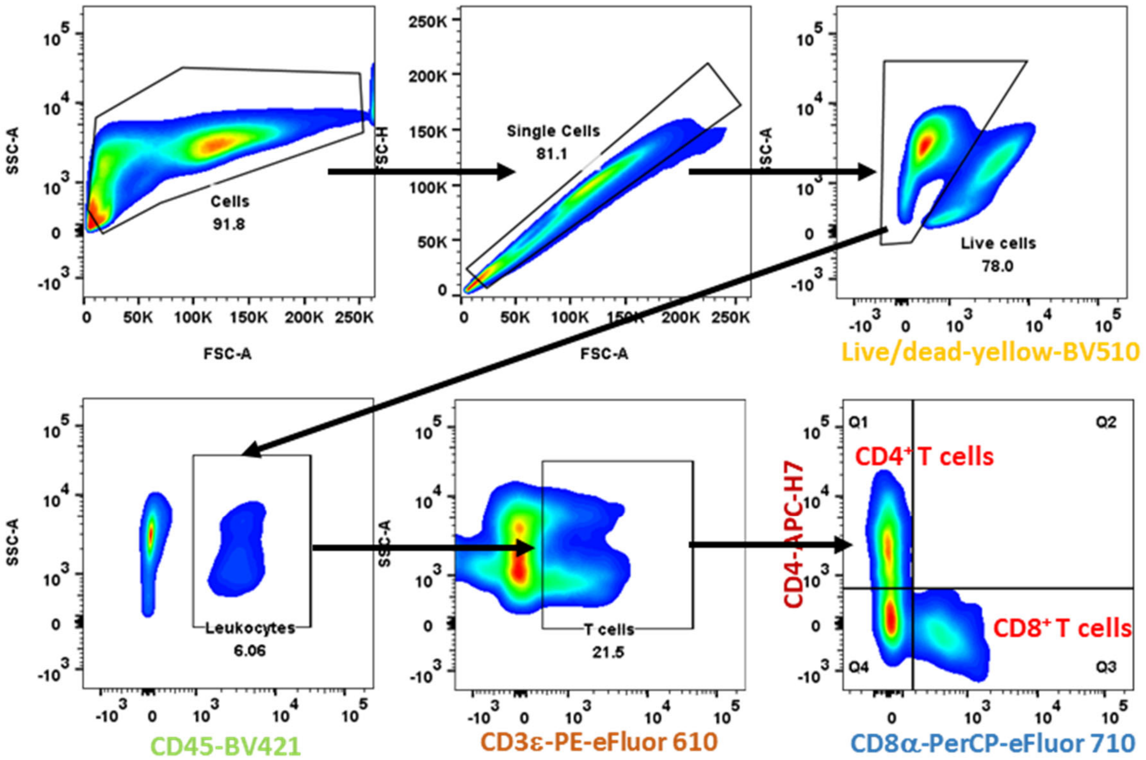

Figure S13 Gating strategies for T cells ($CD45^+CD3e^+$), helper T cells ($CD45^+CD3e^+CD4^+$), cytotoxic T cells ($CD45^+CD3e^+CD8^+$) in Figure 6.

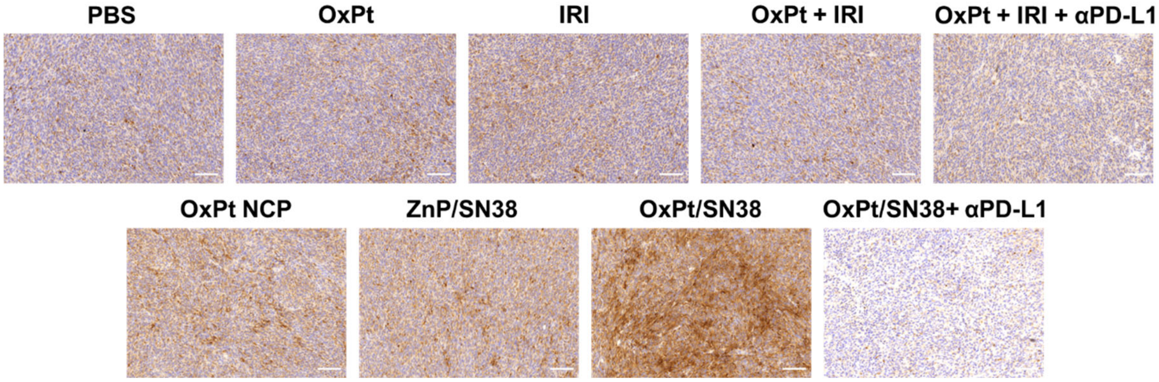

Figure S14 PD-L1 staining of excised MC38 tumors after 8 doses of PBS, OxPt, IRI, OxPt plus IRI, OxPt plus IRI plus αPD-L1, OxPt NCP, ZnP/SN38, OxPt/SN38, or OxPt/SN38 plus αPD-L1. Scale bars are 100 μm.

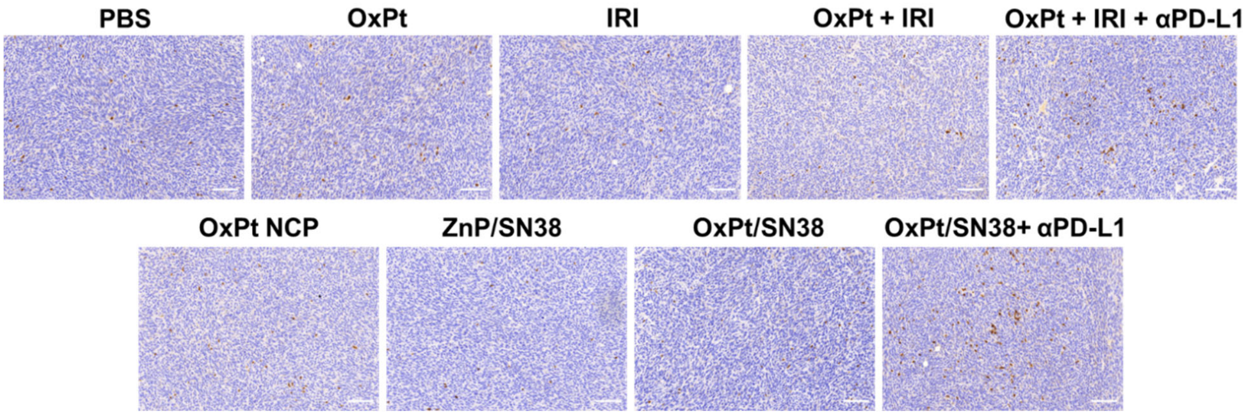

Figure S15 CD3e staining of excised MC38 tumors after 8 doses of PBS, OxPt, IRI, OxPt plus IRI, OxPt plus IRI plus αPD-L1, OxPt NCP, ZnP/SN38, OxPt/SN38, or OxPt/SN38 plus αPD-L1. Scale bars are 100 µm.

Table S1. OxPt and SN38 IC_{50} values (µM) in murine colon cancer cells treated with various formulations

	OxPt	SN38	IRI	SN38-TMS	Chol-SN38	OxPt NCP	ZnP/SN38	OxPt/SN38
CT26	8.23 ± 0.82	0.21 ± 0.06	82.66 ± 7.96	3.14 ± 0.58	8.32 ± 1.52	10.92 ± 1.67	8.68 ± 0.79	1.75 ± 0.23 (3.15 ± 0.41)[a]
MC38	12.51 ± 1.83	0.22 ± 0.07	93.32 ± 8.75	3.54 ± 0.42	10.75 ± 1.73	14.24 ± 1.32	10.86 ± 1.24	2.92 ± 0.44 (5.26 ± 0.79)[a]

[a]The numbers in parentheses refer to SN38 IC50 values.

Table S2. SN38 prodrugs pharmacokinetics of Sprague-Dawley rats following a single intravenous injection of IRI and OxPt/SN38 at a dose of 3.6 mg/kg SN38 equivalent.

Parameter	Unit	IRI	OxPt/SN38[a]
Lambda_z	1/h	0.26 ± 0.06	0.06 ± 0.01
t1/2	h	2.71 ± 1.82	9.74 ± 1.00
Tmax	h	0.08 ± 0.00	1.06 ± 1.68
Cmax	µg/ml	0.83 ± 0.23	161.14 ± 12.45
C0	µg/ml	0.91 ± 0.25	171.05 ± 14.14
Clast_obs/Cmax		0.14 ± 0.06	0.02 ± 0.01
AUC 0-t	µg/ml*h	5.73 ± 2.26	1183.94 ± 28.35
AUC 0-inf_obs	µg/ml*h	6.32 ± 2.45	1215.41 ± 26.26
AUC 0-t/0-inf_obs		0.90 ± 0.15	0.97 ± 0.01
AUMC 0-inf_obs	µg/ml*h^2	237.25 ± 20.53	15503.57 ± 276.38
MRT 0-inf_obs	h	12.62 ± 2.52	12.79 ± 2.51
Vd	(mg/kg)/(µg/ml)	60.70 ± 30.43	0.20 ± 0.02
Cl_obs	(µg/kg)/(µg/ml)/h	556.02 ± 190.16	14.43 ± 1.16

Vss_obs	(mg/kg)/(μg/ml)	59.70 ± 27.19	0.18 ± 0.04

[a]The value was analysis based on Chol-SN38.

Table S3. SN38/SN38-TMS pharmacokinetics of Sprague-Dawley rats following a single intravenous injection of IRI or OxPt/SN38 at a dose of 3.6 mg/kg SN38 equivalent, by non-compartmental analysis.

Parameter	Unit	SN38 from IRI	SN38 from OxPt/SN38	SN38-TMS from OxPt/SN38
Lambda_z	1/h	0.50 ± 0.26	0.16 ± 0.01	0.19 ± 0.07
t1/2	h	1.87 ± 1.34	4.23 ± 1.80	4.03 ± 1.19
Tmax	h	0.08 ± 0.00	0.00 ± 0.00	0.08 ± 0.00
Cmax	ng/ml	227.22 ± 8.68	6.01 ± 0.51	2933.71 ± 1009.37
C0	ng/ml	235.91 ± 10.80	6.08 ± 0.53	3057.37 ± 994.51
Clast_obs/Cmax		0.08 ± 0.01	0.12 ± 0.01	0.05 ± 0.05
AUC 0-t	ng/ml*h	428.78 ± 110.09	47.14 ± 12.05	11388.00 ± 2415.91
AUC 0-inf_obs	ng/ml*h	508.80 ± 209.11	57.16 ± 16.34	12059.63 ± 2282.94
AUC 0-t/0-inf_obs		0.88 ± 0.16	0.82 ± 0.10	1.000 ± 0.000
AUMC 0-inf_obs	ng/ml*h^2	1517.31 ± 147.48	179.60 ± 15.18	63454.68 ± 28385.25
MRT 0-inf_obs	h	2.59 ± 1.56	1.45 ± 0.66	5.07 ± 1.50
Vd	(mg/kg)/(ng/ml)	0.03 ± 0.01	0.39 ± 0.03	3716.38 ± 481.50
Cl_obs	(mg/kg)/(ng/ml)/h	0.02 ± 0.01	0.06 ± 0.01	666.55 ± 137.33
Vss_obs	(mg/kg)/(ng/ml)	0.03 ± 0.01	0.35 ± 0.03	3243.53 ± 396.36

Table S4. Pt pharmacokinetics of Sprague-Dawley rats following a single intravenous injection of OxPt or OxPt/SN38 at a dose of 2 mg OxPt/kg (0.98 mg Pt/kg) by non-compartmental analysis.

Parameter	Unit	OxPt	OxPt/SN38
Lambda_z	1/h	0.78 ± 0.07	0.02 ± 0.01
t1/2	h	0.90 ± 0.32	30.84 ± 4.95
Tmax	h	0.08 ± 0.00	0.22 ± 0.24

Cmax	μg/ml	4.16	±	0.51	17.52	±	2.42
C0	μg/ml	5.03	±	0.58	18.16	±	3.15
Clast_obs/Cmax		0.04	±	0.01	0.25	±	0.10
AUC 0-t	μg/ml*h	22.54	±	6.58	377.10	±	102.39
AUC 0-inf_obs	μg/ml*h	27.41	±	8.26	574.53	±	207.10
AUC 0-t/0-inf_obs		0.82	±	0.02	0.67	±	0.06
AUMC 0-inf_obs	μg/ml*h^2	717.57	±	254.44	25735.89	±	13389.04
MRT 0-inf_obs	h	25.95	±	1.65	42.99	±	7.75
Vd	(mg/kg)/(μg/ml)	2.27	±	0.59	0.08	±	0.02
Cl_obs	(μg/kg)/(μg/ml)/h	77.13	±	20.34	2.40	±	1.23
Vss_obs	(mg/kg)/(μg/ml)	1.99	±	0.47	0.08	±	0.01

Table S5. Tumor $AUC_{0\rightarrow t}$ biodistribution data

	Total Pt (h·μg/mL)	SN38 (h·μg/mL)
Free OxPt	52.1 ± 2.2	--
Irinotecan	--	10.7 ± 1.8
OxPt/SN38	206.7 ± 18.0	50.3 ± 4.3

Table S6. Hematology (neutrophil counts) and key clinical chemistry parameters of female Sprague-Dawley rats treated with OxPt/SN38.[a]

	PBS	OxPt/SN38
neutrophile counts (× 10^3 cells/μL)	0.88 ± 0.31	1.58 ± 0.48
Alanine transaminase (U/L)	26 ± 2	28 ± 4
Aspartate transaminase (U/L)	126 ± 28	109 ± 17
Creatinine (mg/dL)	0.30 ± 0.03	0.30 ± 0.04
Blood urea nitrogen (mg/dL)	16 ± 2	18 ± 2

[a]Female Sprague-Dawleys rats were dosed at 4.0 mg/kg OxPt equivalent and 18.3 mg/kg Chol-SN38 on a once every week schedule for 4 doses.
